# Revealing the Mysteries of Population Mobility Amid the COVID-19 Pandemic in Canada: Comparative Analysis With Internet of Things–Based Thermostat Data and Google Mobility Insights

**DOI:** 10.2196/46903

**Published:** 2024-03-20

**Authors:** Kirti Sundar Sahu, Joel A Dubin, Shannon E Majowicz, Sam Liu, Plinio P Morita

**Affiliations:** 1 School of Public Health Sciences University of Waterloo Waterloo, ON Canada; 2 Department of Statistics and Actuarial Science University of Waterloo Waterloo, ON Canada; 3 School of Exercise Science, Physical and Health Education University of Victoria Victoria, BC Canada; 4 Institute of Health Policy, Management, and Evaluation University of Toronto Toronto, ON Canada; 5 Research Institute of Aging University of Waterloo Waterloo, ON Canada; 6 Department of Systems Design Engineering University of Waterloo Waterloo, ON Canada; 7 eHealth Innovation University Health Network Toronto, ON Canada

**Keywords:** population-level health indicators, internet of things, public health surveillance, mobility, risk factors, chronic diseases, chronic, risk, surveillance, mobility, movement, sensor, population

## Abstract

**Background:**

The COVID-19 pandemic necessitated public health policies to limit human mobility and curb infection spread. Human mobility, which is often underestimated, plays a pivotal role in health outcomes, impacting both infectious and chronic diseases. Collecting precise mobility data is vital for understanding human behavior and informing public health strategies. Google’s GPS-based location tracking, which is compiled in Google Mobility Reports, became the gold standard for monitoring outdoor mobility during the pandemic. However, indoor mobility remains underexplored.

**Objective:**

This study investigates in-home mobility data from ecobee’s smart thermostats in Canada (February 2020 to February 2021) and compares it directly with Google’s residential mobility data. By assessing the suitability of smart thermostat data, we aim to shed light on indoor mobility patterns, contributing valuable insights to public health research and strategies.

**Methods:**

Motion sensor data were acquired from the ecobee “Donate Your Data” initiative via Google’s BigQuery cloud platform. Concurrently, residential mobility data were sourced from the Google Mobility Report. This study centered on 4 Canadian provinces—Ontario, Quebec, Alberta, and British Columbia—during the period from February 15, 2020, to February 14, 2021. Data processing, analysis, and visualization were conducted on the Microsoft Azure platform using Python (Python Software Foundation) and R programming languages (R Foundation for Statistical Computing). Our investigation involved assessing changes in mobility relative to the baseline in both data sets, with the strength of this relationship assessed using Pearson and Spearman correlation coefficients. We scrutinized daily, weekly, and monthly variations in mobility patterns across the data sets and performed anomaly detection for further insights.

**Results:**

The results revealed noteworthy week-to-week and month-to-month shifts in population mobility within the chosen provinces, aligning with pandemic-driven policy adjustments. Notably, the ecobee data exhibited a robust correlation with Google’s data set. Examination of Google’s daily patterns detected more pronounced mobility fluctuations during weekdays, a trend not mirrored in the ecobee data. Anomaly detection successfully identified substantial mobility deviations coinciding with policy modifications and cultural events.

**Conclusions:**

This study’s findings illustrate the substantial influence of the Canadian stay-at-home and work-from-home policies on population mobility. This impact was discernible through both Google’s out-of-house residential mobility data and ecobee’s in-house smart thermostat data. As such, we deduce that smart thermostats represent a valid tool for facilitating intelligent monitoring of population mobility in response to policy-driven shifts.

## Introduction

The dynamics of human mobility are currently undergoing a remarkable transition. Rapid global urbanization, sedentary lifestyles, infectious diseases, air pollution, and climate change are some of the factors driving shifts in human mobility. Whether examined at the individual [1,2] or population level [3], human mobility patterns are associated with multiple public health and social issues. Dwindling mobility patterns are linked to chronic diseases including dementia and age-related physical decline. For infectious diseases, individual mobility is linked to the spread of infections such as COVID-19. Human mobility data can therefore be an effective tool for comprehending the complexities of human health and behavior [4].

The COVID-19 pandemic has served as an ideal case study to develop tools and knowledge in the field of human mobility research [5]. The World Health Organization declared COVID-19 an international public health emergency in late January 2020 and subsequently declared it a pandemic on March 11, 2020. To curb the spread of COVID-19 and avoid overwhelming health care institutions [6], many countries implemented restrictions on human mobility including social distancing, self-isolation, closure of nonessential services, work-from-home policies, and travel restrictions [7,8]. In the early stages of the pandemic, digital data sources found that the decrease in human mobility was closely paralleled by a reduction in the incidence of COVID-19 [1,7,9-15]. Thus, the COVID-19 pandemic provides an ideal opportunity to measure and extract meaning from human mobility and how it is affected by restrictive policies.

The study of human mobility has historically been retrospective with limited study participants. Interestingly, it was the technological advancements in smartphones and wearable devices that have had the largest impact on the field [16]. These innovations now provide direct access to human location, trajectories, opinions, and interactions [17]. Human mobility can now be tracked passively in real time from various sources: GPS-enabled smartphones, texts or photos via photo-sharing platforms like Twitter (X Corp) and Flickr (Altaba, Ludicorp), geolocation-enabled internet posts, public transport cards, satellite, flight traffic, and even credit card transactions. The data collected have a previously unprecedented level of detail, immediacy, and precision. These large data sets, equivalent to “big data” in volumes, are now being analyzed to describe human movement patterns, characteristics (such as sleep, stress, and activity), and interactions.

Google Maps is the most popular navigation app in the United States and Canada. The app surpassed 23 million downloads in 2020 with 154.4 million monthly users. Google passively generates and collects over 20 million pieces of mobility data per day. The information has now been made available to researchers and policy makers through Google’s open-source “COVID-19 Community Mobility Reports” [18]. The reported geographic movement, called macromobility, is grouped by category: retail and recreation, grocery and drug stores, parks, transit stations, workplaces, and residences [18]. While macromobility data are readily available through sources such as Google’s “COVID-19 Community Mobility Reports,” our understanding of micromobility at the population level remains limited. This study aims to fill this gap by analyzing smart thermostat data, thereby providing a more comprehensive picture of human mobility patterns. This study leverages the unprecedented access to real-time human mobility data provided by smart thermostats, a source that has not been extensively used in previous research. The high level of detail, immediacy, and precision of these data allows for a more granular understanding of human mobility patterns, particularly in-house mobility (micromobility), which has been less explored compared to macromobility. Unlike Google’s macromobility data, smart thermostat data can provide insights into in-house mobility patterns. This is particularly relevant in the context of the COVID-19 pandemic, where stay-at-home orders and work-from-home policies have significantly altered in-house mobility patterns.

Modular smart home thermostats offer a novel source of data on in-house human behaviors [19,20]. Not only can they report on indoor temperature, humidity, and air quality, but embedded motion sensors can capture mobility within the home. More than 90% of Canadian households had thermostats in 2018, with most opting for programmable thermostats. Smart thermostats, often known as internet of things (IoT) devices, are a type of programmable thermostat that may be connected to the internet. ecobee has the second-highest market share for smart thermostats in Canada. ecobee has a program called Donate Your Data (DYD), in which subscribers can opt to make their anonymized data available for research purposes. DYD currently has 1 million users including over 172,000 households in Canada. Here, we sought to compare mobility data obtained from ecobee smart thermostats to the “gold standard” mobility data from Google during the COVID-19 pandemic in Canada. We explored day-by-day, week-by-week, and month-by-month seasonality patterns and applied anomaly detection to both data sets.

## Methods

### Data Sources

Google Mobility data were collected for each province in Canada from February 15, 2020, to February 14, 2021 [18]. Google Maps uses aggregated and anonymized data to determine the daily total number of visits to specific destinations (Textbox 1) visited by individuals who have enabled their location history [18]. Daily values are compiled across individuals and are compared to the baseline value for that day of the week to determine changes in mobility. The baseline is the median of corresponding days over the 5 weeks from January 3 to February 6, 2020 [18]. Out of the 6 categories, we focused on residential data. We curated mobility data from ecobee’s DYD program from February 15, 2020, to February 14, 2021, to align with the Google Mobility Report publication dates. ecobee’s smart thermostats record in-house mobility via motion sensors. The DYD data set supplied by ecobee is hosted in Google BigQuery. This study analyzed data from 4 provinces in Canada: Ontario, Alberta, Quebec, and British Columbia. This included 12,252 ecobee households. These 4 provinces constitute approximately 87% of the Canadian population [21]. The reliability and validity of the ecobee data set were rigorously assessed in our prior studies, as detailed in our previously published research [20,22,23]. Information regarding Canadian policy implementation dates across provinces was obtained from a dedicated platform managed by the Canadian Government to share COVID-19–related information regularly [24].

**Google mobility data categories and their description as described on the website [18].**

**Grocery and pharmacy**

- “Mobility trends for places like grocery markets, food warehouses, farmers markets, specialty food shops, drug stores, and pharmacies.”

**Parks**

- “Mobility trends for places like local parks, national parks, public beaches, marinas, dog parks, plazas, and public gardens.”

**Transit stations**

- “Mobility trends for places like public transport hubs such as subway, bus, and train stations.”

**Retail and recreation**

- “Mobility trends for places like restaurants, cafes, shopping centers, theme parks, museums, libraries, and movie theatres.”

**Residential**

- “Mobility trends for places of residence.”

**Workplaces**

- “Mobility trends for places of work.”

### Ethical Considerations

Per ethical research standards, this study exclusively used secondary data, and therefore, no additional consent from human subjects was necessary for this research. The original informed consent procedures or institutional review board approvals for primary data collection explicitly permitted the secondary analysis conducted in this study. Furthermore, robust privacy and confidentiality safeguards were implemented to ensure the anonymity and deidentification of study data. Ethics approval for this study was duly obtained from the University of Waterloo Office of Research Ethics (#31377), attesting to our commitment to upholding the highest ethical standards in research.

### Data Preparation

ecobee’s data were transferred from Google’s BigQuery to the Microsoft Azure cloud services platform [25]. Data were prepared using Azure Databricks data analytics platform and Jupyter Notebook (Jupyter Team) using Python; detailed explanation of the whole process has been mentioned in our previous studies [20,23]. Data cleaning, analysis, and visualization were done in R studio (version 1.4.1106; Posit Software, PBC) with R software (version 4.0.5; The R Foundation) and data analysis packages *tidyverse* [26] and *timetk* [27].

Using ecobee data, we established a baseline mobility value akin to the Google data preparation approach detailed earlier for seamless comparison. To ascertain daily in-house mobility, we computed the total number of activated sensors within each 24-hour timeframe by summing all sensor statuses. A table containing date records alongside the average count of activated sensors per date was then saved as a CSV file.

As the timestamp of the DYD data set was in Coordinated Universal Time format, the time zones in the data set were converted by locating time zone information from the geolocation of the households in the metadata. In Quebec and Alberta, all the cities were in the same time zone. In the province of Ontario, 6 cities have different time zones than EST: namely, Drayton, Kenora, Kenora-Unorganized, Mitchell, Red Lake, and Sioux Lookout cities (1% of the DYD data set for Ontario). For British Columbia, 28 households were from different time zones than Pacific Standard Time and were excluded from the analysis. Once cleaned for the time zone, time-series data analysis was performed on the adjusted data on the households included in this study. These numbers are presented in Table 1.

**Table 1:** The number of households selected for the analysis by province.

Location	Households before time zone cleaning, n	Households excluded for time zone difference, n	Households after time zone cleaning, n	The proportion of household data by province, n/N (%)
Canada	21,690	—^a^	21,690	—
Ontario	7145	11	7134	7134/21,690 (32.9)
Alberta	3989	0	3989	3989/21,690 (18.4)
British Columbia	449	28	421	421/21,690 (1.9)
Quebec	708	0	708	708/21,690 (3.2)

^a^Not available.

### Data Analysis

To assess mobility variations, we aggregated daily province-level movement into weekly, monthly, and day-of-the-week periods and compared them to the respective baselines in both data sets. Time series plots were generated for each province using Google’s residential and ecobee mobility data. The statistical significance of the relationship between the 2 data sources was determined through Pearson and Spearman correlation coefficients [28].

We conducted seasonal diagnostic tests using the *timetk* [27] package in R software, tailored for time-series data analysis. Distinct approaches were employed to investigate daily, weekly, and monthly seasonality. To assess statistical significance [29] 1-way ANOVA was used.

For anomaly detection, we initially conducted seasonal and trend decomposition using the Loess method [27,30,31]. After removing the trend and seasonality components, we performed anomaly detection on the residual data. Anomalies were identified based on the IQR, specifically the difference between the 75th and 25th percentiles, establishing the distribution of the remaining data. Default boundaries were set at 3× above or below the IQR, designating values beyond these limits as anomalies.

To examine data granularity, we analyzed mobility data by days of the week for both Google and ecobee data sets. Additionally, we aggregated mobility data into 1-week intervals for all 4 provinces to explore seasonality in mobility changes following the onset of the COVID-19 pandemic.

## Results

### Positive Association Between Google and ecobee Mobility Data

**Figure 1:**
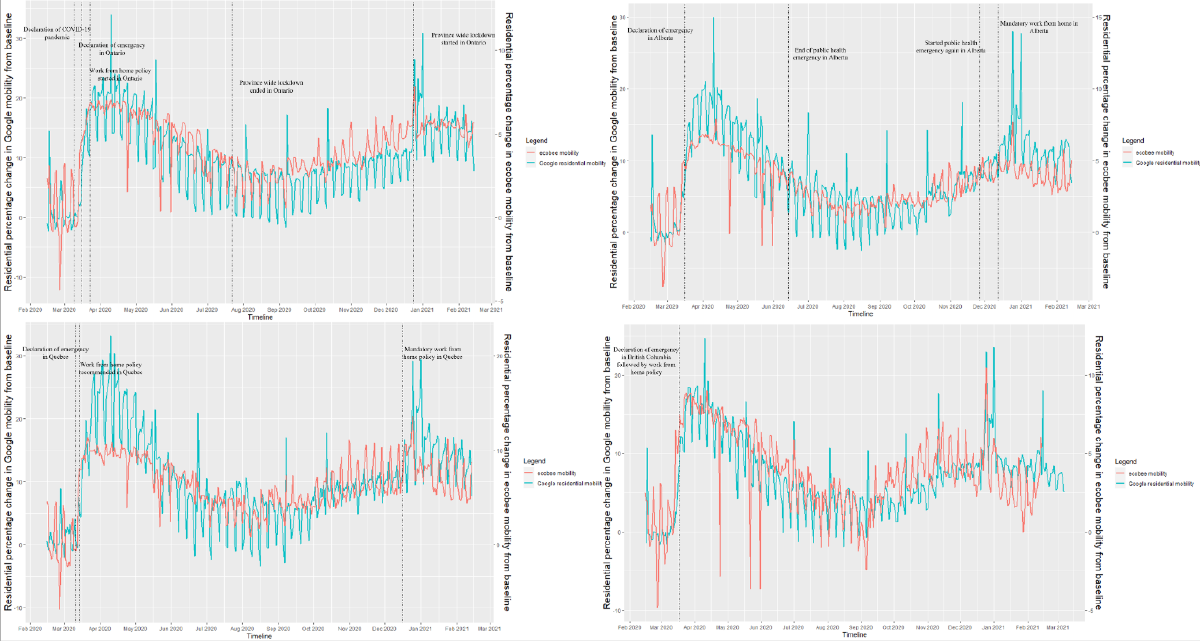
Association between Google residential mobility and ecobee mobility for (A) Ontario, (B) Alberta, (C) Quebec, and (D) British Columbia, Canada. A high resolution version is available in Multimedia Appendix 1.

The comparison of Google residential mobility data and ecobee mobility data across each province over a year revealed a positive association (Figure 1). Notably, the Google mobility data exhibited a recurrent weekly pattern, which was closely mirrored by the ecobee data (Figure 1). Both data sources exhibited a significant uptick in residential mobility, both indoors and outdoors, commencing around March 11, 2020, aligning with the declaration of the COVID-19 pandemic in Canada. It is worth noting that given Canada’s provincial health regulation framework [32], the official implementation dates of pandemic-related policies varied among the 4 provinces under investigation (March 17, 2020, for Ontario; March 16, 2020, for Quebec; March 17, 2020, for Alberta; and March 19, 2020, for British Columbia).

All provinces exhibited a notable increase in the percentage of mobility change coinciding with the commencement of the pandemic, as indicated by both Google and ecobee mobility data. Subsequently, from March 2020 to September 2020, a gradual decline in this trend was observed across all provinces in both data sets. Notably, a subsequent surge in residential mobility was linked to the onset of the second pandemic wave and the reinstatement of pandemic-related stay-at-home measures on specific dates (December 26, 2020, for Ontario; December 17, 2020, for Quebec; December 13, 2020, for Alberta; and October 19, 2020, for British Columbia).

**Figure 2:**
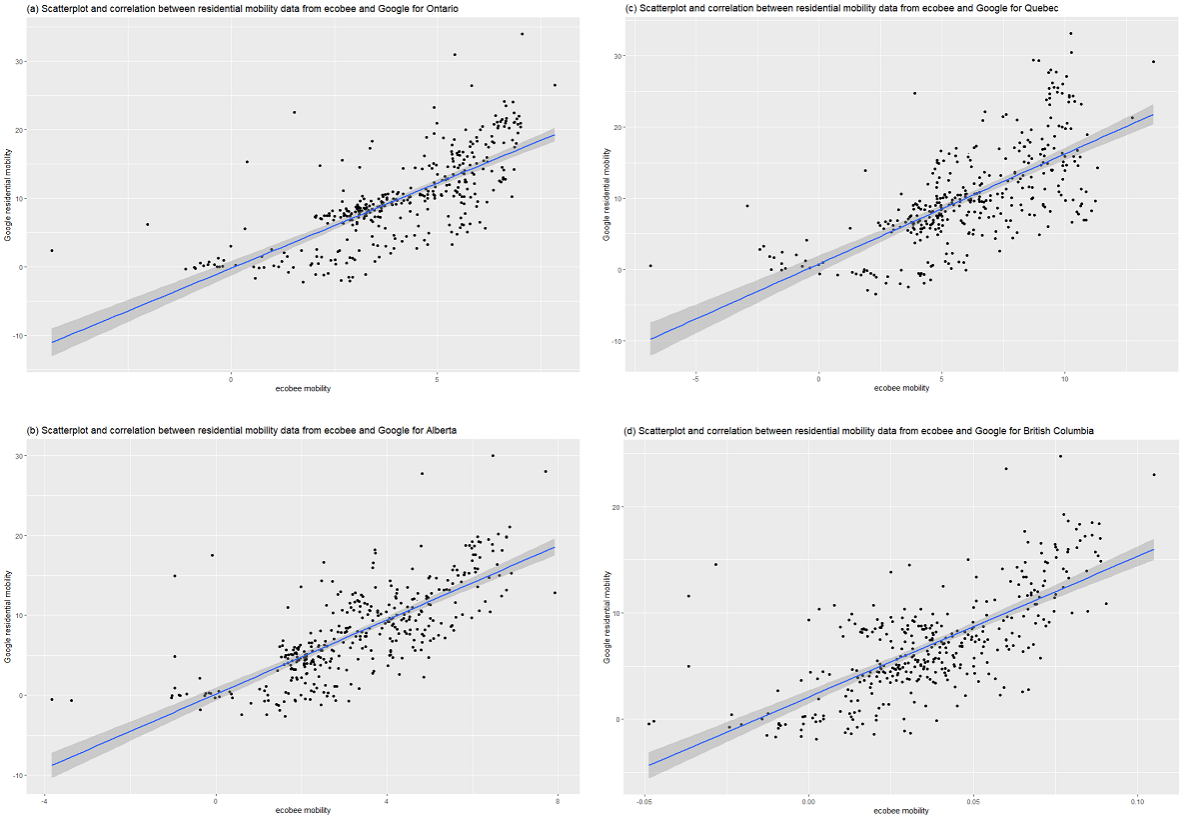
Correlation between residential mobility data from ecobee and Google for the province of (A) Ontario, (B) Alberta, (C) Quebec, and (D) British Columbia, Canada. A high resolution version is available in Multimedia Appendix 1.

For each province, we calculated the correlation between Google’s residential mobility data and ecobee’s mobility data. The trend line displayed a linear relationship between the 2 data sets (see Figure 2). Pearson and Spearman correlation coefficients revealed a statistically significant association, ranging from 0.67 to 0.73 (refer to Table 2). Consequently, the data obtained from ecobee’s smart home thermostat are demonstrably equivalent to Google’s mobility data.

**Table 2:** Correlation between Google and ecobee mobility data for Ontario, Alberta, Quebec, and British Columbia.

Province	Households, n	Pearson correlation coefficient (95% CI)	Spearman rank correlation
Ontario	7134	0.73 (0.67-0.77)	0.75
Alberta	3989	0.73 (0.69-0.78)	0.76
Quebec	708	0.67 (0.61-0.73)	0.70
British Columbia	421	0.69 (0.64-0.74)	0.63

### Variations in Mobility by Day of the Week

**Figure 3:**
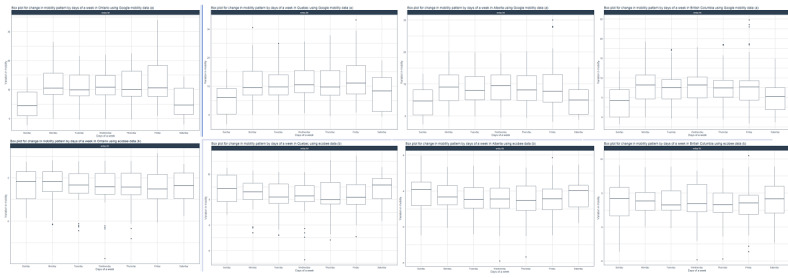
Analysis of the (A) Google residential and (B) ecobee mobility data in days of a week in Ontario, Quebec, Alberta, and British Columbia. A high resolution version is available in Multimedia Appendix 1.

There was a significant difference in daily mobility patterns across all 4 provinces when examining the Google residential mobility data set (Figure 3). Greater mobility changes were observed on the weekdays compared to weekends. Further, a 1-way ANOVA test (Table 3) showed that the day of the week had a statistically significant impact on residential mobility for all 4 provinces (Google; all *P*<.001). On the contrary, these differences in daily mobility were only observed in Quebec when we used ecobee’s mobility data.

**Table 3:** The ANOVA test compares the day of the week’s impact on Google and ecobee mobility for the 4 provinces of Canada.

Province		Google				ecobee		
		Sum of squares	*F* test (*df*)	*P* value	Sum of squares		*F* test	*P* value
**Ontario**			17.86 (6, 358)	<.001			0.458 (6, 358)	.84
	Weekday	3467			10			
	Residual	11,579			1306			
**Quebec**			9.357 (6, 358)	<.001			2.364 (6, 358)	.03
	Weekday	2426			130			
	Residual	15,471			3278			
**Alberta**			8.223 (6, 358)	<.001			1.123 (6, 358)	.35
	Weekday	1456			22.9			
	Residual	10,566			1216			
**British Columbia**			6.955 (6, 358)	<.001			0.371 (6, 358)	.90
	Weekday	897			14.9			
	Residual	7673			2404			

### Monthly Variations in Mobility

**Figure 4:**
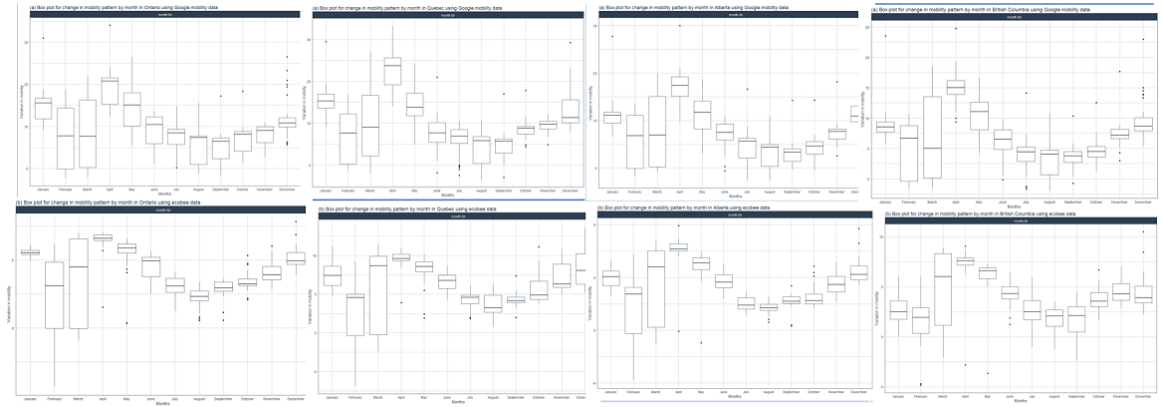
Analysis of the (A) Google residential and (B) ecobee mobility data in month-by-month for Ontario, Alberta, Quebec, and British Columbia. A high resolution version is available in Multimedia Appendix 1.

To deepen our understanding of the pandemic-related changes in population behavior, we aggregated the Google and ecobee data to analyze month-by-month mobility changes. Interestingly, in contrast to the differences observed between Google and ecobee mobility data for days of the week, month-by-month patterns were similar between the 2 data sets (Figure 4). Across all provinces, the change in mobility above baseline spiked in April 2020 consistent with the implementation of COVID-19 policies to curb social mobility. Further, the variability within the data, specifically for ecobee data, was reduced drastically from April 2020 onwards. The increase in residential and in-home mobility slowly declined from April 2020 to September 2020. A subsequent rise in mobility from October 2020 to December 2020 corresponded to the second pandemic wave and the reimplementation of pandemic-related stay-at-home policies. Furthermore, 1-way ANOVA test showed a statistically significant change in both residential (Google) and in-house (ecobee) month-by-month mobility across all 4 provinces (all *P*<.001; Table 4).

**Table 4:** The ANOVA test compares month-by-month impact on Google and ecobee mobility for the 4 provinces of Canada.

Province		Google				ecobee		
		Sum of squares	*F* test (*df*)	*P* value	Sum of squares		*F* test (*df*)	*P* value
**Ontario**			23.65 (11, 353)	<.001			26.18 (11, 353)	<.001
	Month	6383			591.4			
	Residual	8663			724.8			
**Quebec**			36.64 (11, 353)	<.001			21.61 (11, 353)	<.001
	Month	9541			1372			
	Residual	8357			2037			
**Alberta**			31.16 (11, 353)	<.001			30.76 (11, 353)	<.001
	Month	5923			606.4			
	Residual	6099			632.7			
**British Columbia**			28.83 (11, 353)	<.001			24.49 (11, 353)	<.001
	Month	4062			1047			
	Residual	4508			1372			

### Week-by-Week Mobility Changes

**Figure 5:**
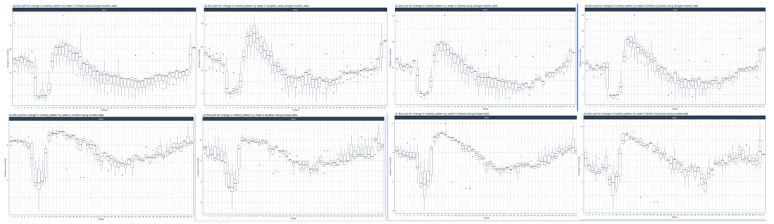
Analysis of the (A) Google residential and (B) ecobee mobility data in week-by-week for Ontario, Alberta, Quebec, and British Columbia. A high resolution version is available in Multimedia Appendix 1.

Beginning in February 2020, the initial 5 to 6 weeks had a similar level of mobility across all 4 provinces (Figure 5). Google residential mobility declined from week 7 to week 10. Although there was a high level of data variability at week 7, this was lost in subsequent weeks. ecobee in-house mobility followed a similar pattern but with a lag period of 1 week and a large degree of variability. Beginning at week 12 (corresponding to the week of March 16, 2020), Google residential data witnessed a sharp spike in residential mobility across all 4 provinces. This timing correlates to the date the pandemic was declared in Canada. A similar trend was observed for the ecobee data, however, mobility appeared to increase starting in week 11. Similar to the trends observed in month-by-month data (Figure 4), the elevated mobility steadily declined toward week 25 and stabilized above baseline until approximately week 40 (Figure 5). There was a subsequent steady increase in mobility from week 40 until the end of the year corresponding to the timing of the second wave of the pandemic in Canada. The ecobee mobility data showed a similar trend with the exception that the decline in mobility took place over a longer period and with a shorter period of stabilization before rising again. An ANOVA analysis for the week-by-week mobility data showed a statistically significant difference between weeks for both data sets and for all 4 provinces (all *P*<.001; Table 5).

**Table 5:** The ANOVA test comparing the week-by-week impact on Google and ecobee mobility for the 4 provinces of Canada.

Province			Google							ecobee				
			Sum of squares		*F* test (*df*)		*P* value		Sum of squares			*F* test (*df*)		*P* value
**Ontario**					12.4 (52, 312)		<.001					18.43 (52, 312)		<.001
	Week	10,140						992.9						
	Residual	4906						323.2						
**Quebec**					18.88 (52, 312)		<.001					13.79 (52, 312)		<.001
	Week	13,582						2375						
	Residual	4316						1034						
**Alberta**					17.57 (52, 312)		<.001					17.84 (52, 312)		<.001
	Week	8961						927.3						
	Residual	3061						311.9						
**British Columbia**					19.49 (52, 311)		<.001					13.53 (52, 312)		<.001
	Week	6558						1675.8						
	Residual	2012						742.9						

### Social Mobility Policy Restrictions and Festive Periods Create Mobility Anomalies

**Figure 6:**
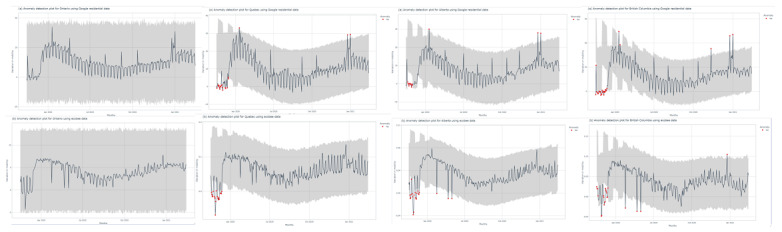
Analysis of the (A) Google residential and (B) ecobee mobility data in days of a week for Ontario, Alberta, Quebec, and British Columbia. A high resolution version is available in Multimedia Appendix 1.

To determine whether Google and ecobee data analysis could pick out behavioral anomalies in mobility associated with policy changes, we performed an anomaly detection analysis (Figure 6). Neither Google residential data nor ecobee in-house mobility data showed any anomaly within 2020 for Ontario. For Quebec, Alberta, and British Columbia, anomalies were found at the beginning period in both the Google and ecobee data. Notably, these corresponded with the dates of COVID-19–related policy changes. In Alberta and British Columbia, ecobee’s in-house mobility analysis captured anomalies in May and June 2020, which correspond to phase-wise reopening plans and the lifting of social restrictions. Interestingly, Google residential data were able to capture anomalies corresponding to festive periods such as Christmas and New Year’s Eve in Quebec, Alberta, and British Columbia. These anomalies were not seen in the ecobee data. Overall, these results demonstrate the ability of Google’s residential data and ecobee’s thermostat data to capture notable shifts in population behavior as a result of policy changes and cultural festivities.

## Discussion

This study aimed to investigate the potential application of in-house mobility data obtained from ecobee sensors in comparison to residential Google mobility data. The most significant differences between data sets were observed, particularly when mobility was analyzed by the day of the week. While Google’s residential data exhibited a significant correlation with the day of the week across all 4 provinces, ecobee’s data showed a significant impact only in Quebec. However, when scrutinized on a monthly and weekly basis, consistent findings were observed across all provinces and data sets. A notable surge in in-house and residential mobility occurred between March and April 2020, coinciding with the initiation of pandemic-related policy changes in Canada. These mobility shifts gradually diminished until September 2020, and the onset of the second wave of the pandemic, coupled with the reinstatement of social policies, corresponded with increased mobility from October to December 2020. In summary, a statistically significant association between the 2 data sets was identified. Anomaly detection analysis provided evidence supporting the capability of both data sets to detect deviations in population mobility, capturing events such as emergency declarations, reopening phases, and festive days such as December 25 (Christmas) and January 1 (New Year’s Eve). These findings underscore the benefits of employing public health surveillance mechanisms, especially during health emergencies of pandemic proportions.

Historically, monitoring individual mobility has been hampered by insufficient sample sizes, difficulty in collecting data, recall bias, and privacy concerns [33-35]. The advent of smartphones, wearable devices, and the IoT has paved the way for researchers to capture individual geolocation and movement within the home (micromobility) [36-40]. For both micro- and macromobility, it is necessary to integrate data from multiple sources [41]. Thermostat-based IoT data, such as the ecobee DYD data reported here, can provide new opportunities for calculating population-level mobility indicators. IoT is a modern, passive sensing tool that can quantify in-house movement. Understanding in-house movement is particularly relevant given that people now spend more than 80% of their time indoors [4,42]. The use of these large data sets, combined with geographical data and timestamps [16,17], has the potential to unravel different dimensions of human behavior and lifestyle. In-house mobility data can measure changes in sleep [5,43], physical activity [44], sedentary behaviors [45-47], and movement patterns [48] with greater detail and granularity. Motion sensors are a next-generation tool with a wealth of opportunities for application in the field of public health. Integrated into smart cities, motion sensors have the power to detect overall human mobility from various sources. Data collection will enable effective planning and implementation of responsive and preventative public health strategies [37].

Monitoring real-time population mobility is important in public health as it plays a significant role in both chronic and infectious diseases. A dwindling mobility pattern is a predisposition to various chronic diseases including dementia and age-related physical decline. For infectious diseases, individual mobility is directly proportional to the transmission of infections such as COVID-19 [49]. Although the “stay-at-home” and “work-from-home” strategies were promoted globally to curb the spread of COVID-19, it is still unclear to what extent individuals complied with policy restrictions. Mobility data analysis has the potential to provide real-time information about the impact of such policies on individual and population behavior [12,50,51].

However, a notable limitation of our study was the absence of sociodemographic information in the ecobee data set. Consequently, our analysis was confined to spatiotemporal dimensions, and we were unable to examine the impact of sociodemographic features, including age, gender, and occupation. Varied perceptions surrounding infectious diseases, vaccinations, social mobility, and government policies across cultural and socioeconomic groups highlight the need for caution when generalizing the results [52]. Although the data were collected across 4 Canadian provinces, encompassing 87% of the population, the demographic represented by those with smart home thermostats likely skews toward a specific group—namely, young, tech-savvy individuals with higher socioeconomic status, who may be more inclined to work from home. Therefore, caution should be exercised in generalizing the reported results. Additionally, challenges arise in separating mobility patterns in multiperson households and eliminating sensor activation due to factors such as animals, rapid airflow, or other noises.

In conclusion, the real-time monitoring of population-level mobility using smartphones and IoT sensors has emerged as a recent development in public health, primarily in response to the COVID-19 pandemic. This study investigates the utility of IoT-based mobility data, specifically from smart thermostats, for assessing individual mobility within the context of social isolation policies. The findings demonstrate a close alignment between thermostat mobility data and the data presented in the Google Mobility Report, affirming its value as a mobility monitoring tool. The acquisition of real-time mobility data from smart thermostats has the potential to enhance our understanding of the intricate social determinants of health, providing valuable insights for the formulation of public health policies.

## Appendix

Multimedia Appendix 1 High resolution images of Figures 1-6.

## Abbreviations

DYD: Donate Your Data
IoT: internet of things

## Data Availability

The Google mobility data can be readily accessed by the public [18]. In contrast, access to ecobee data is restricted to researchers and scientists through a data-sharing agreement. If you are interested in accessing ecobee data, please email the research division of ecobee.
