# Supplementary figures and images for: Revealing the Mysteries of Population Mobility Amid the COVID-19 Pandemic in Canada: Comparative Analysis With Internet of Things–Based Thermostat Data and Google Mobility Insights
Source: JMIR Public Health Surveill. 2024 Mar 20;10:e46903. doi: 10.2196/46903 (PMC10993118; doi:10.2196/46903)

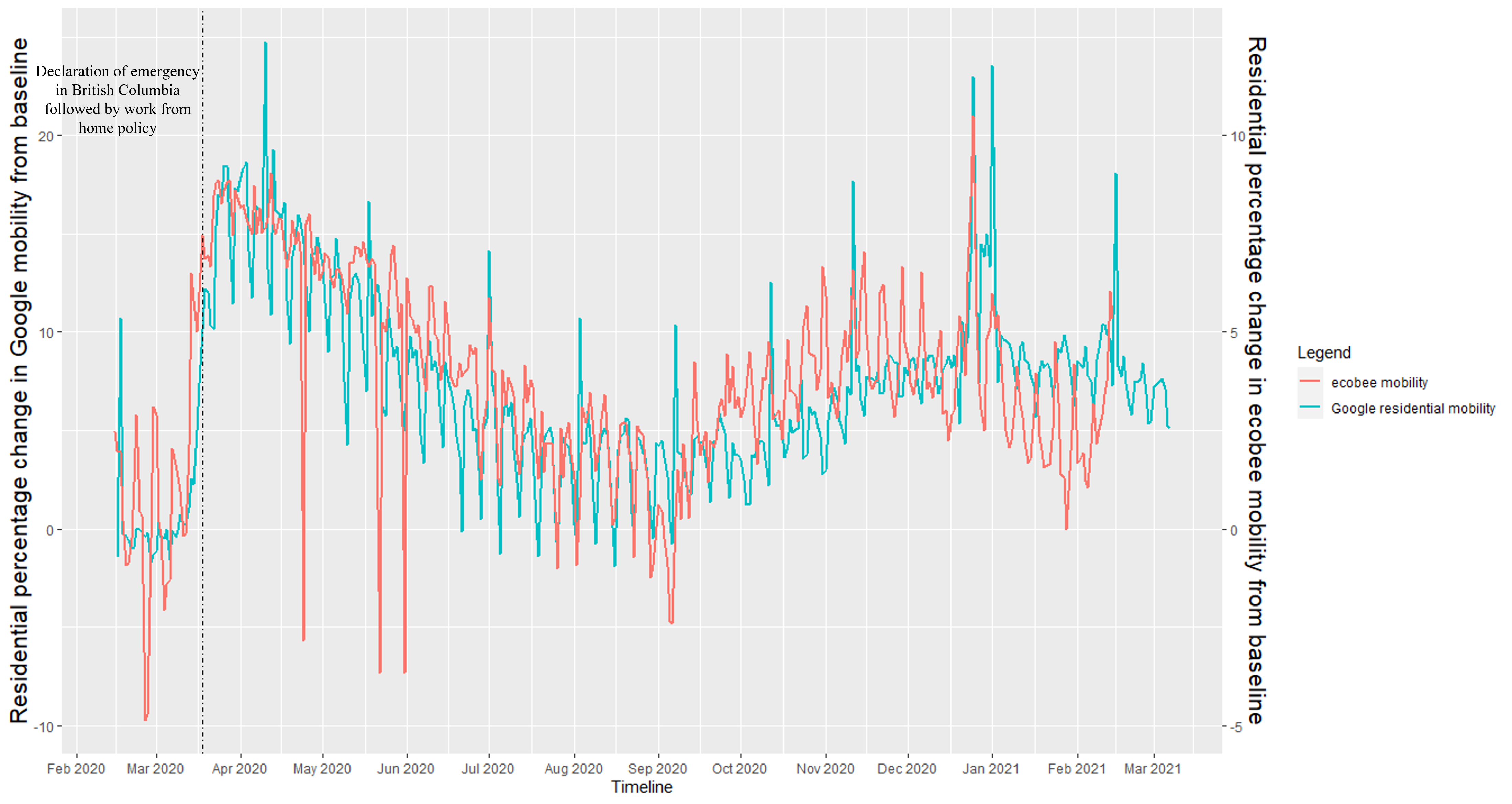

Supplement: Multimedia Appendix 1 [file publichealth_v10i1e46903_app1.zip › Figures 1-6 MA1/Figure-1/(D) British Columbia.png]

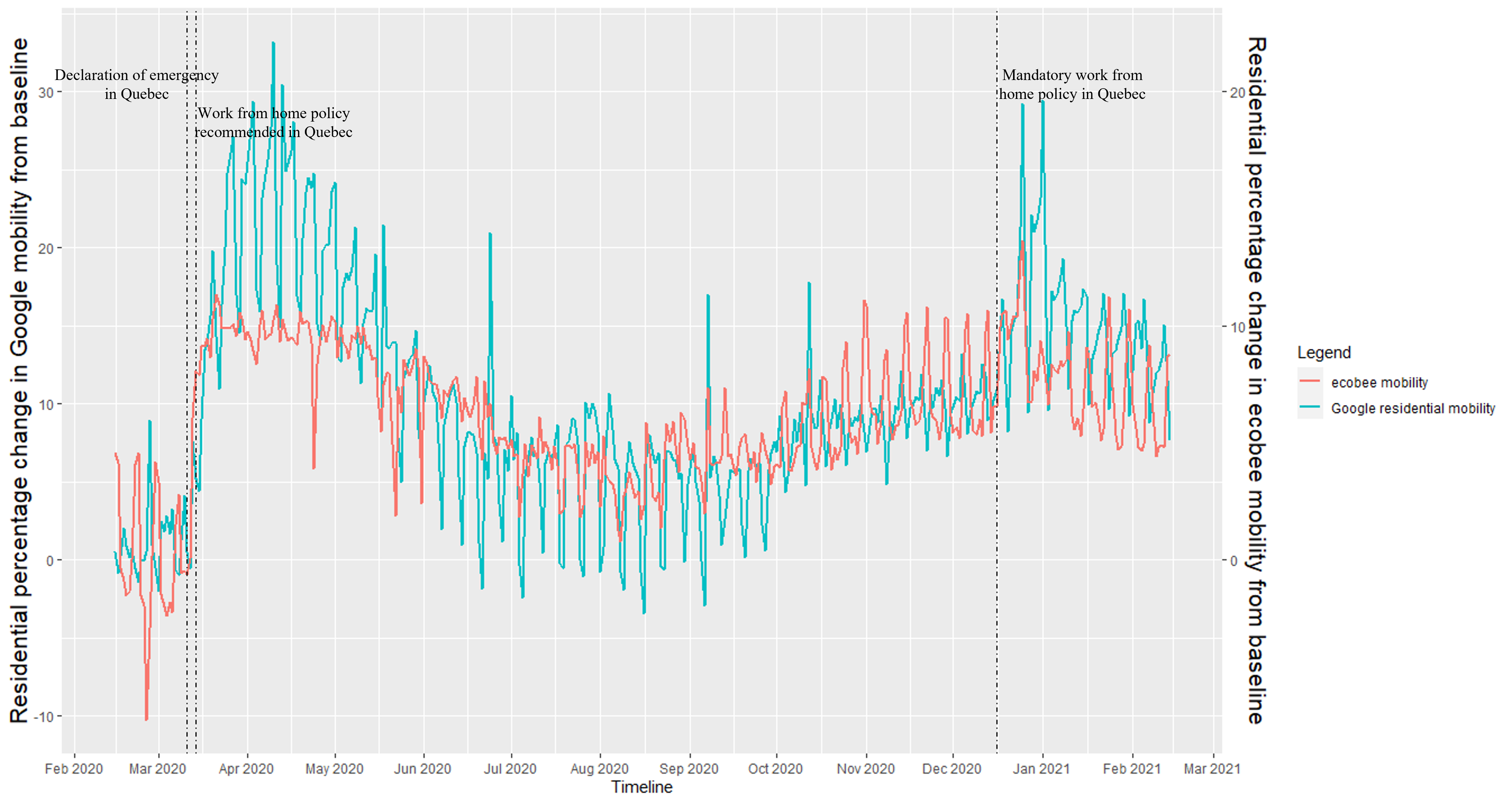

Supplement: Multimedia Appendix 1 [file publichealth_v10i1e46903_app1.zip › Figures 1-6 MA1/Figure-1/(C) Quebec.png]

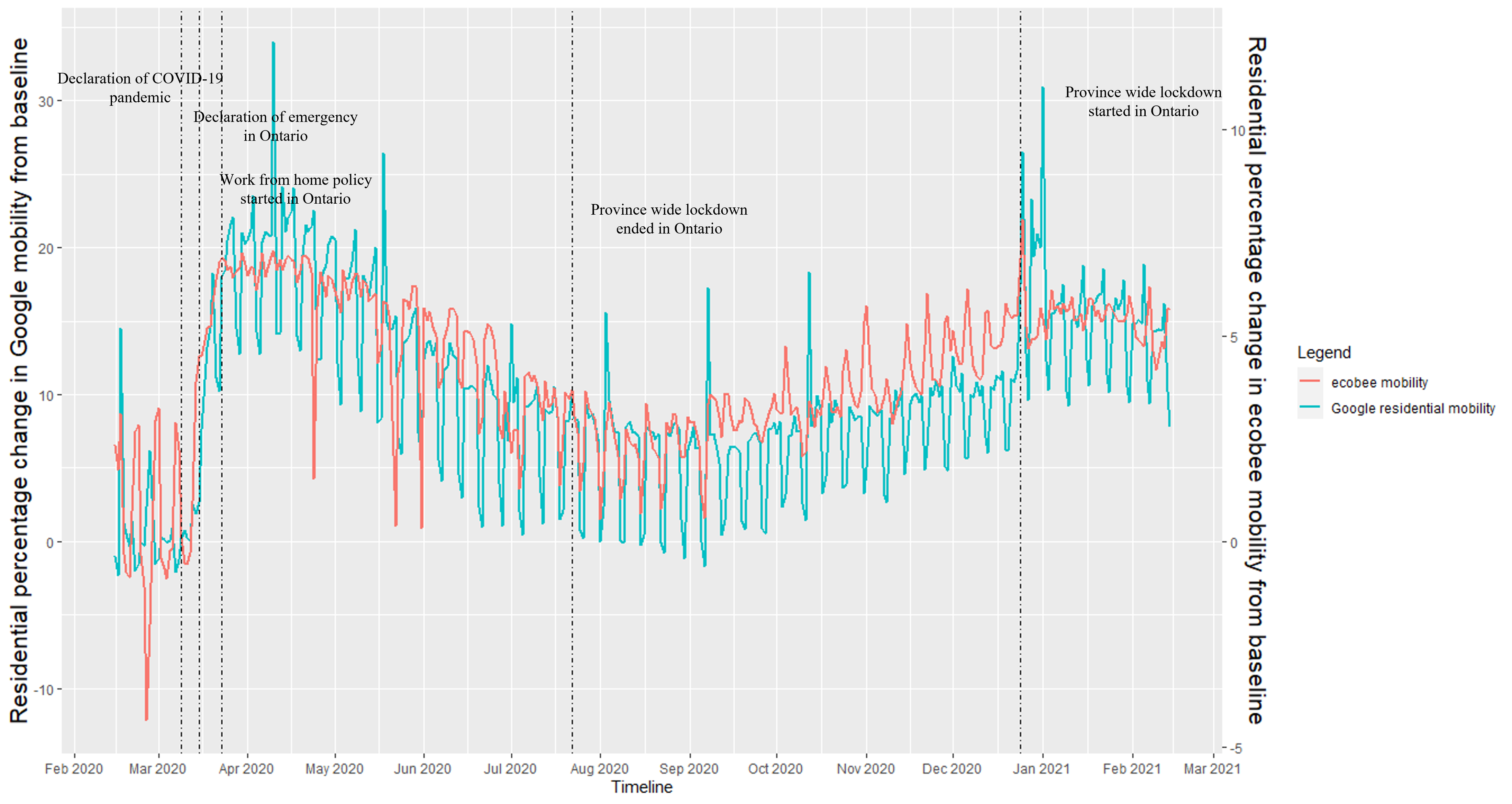

Supplement: Multimedia Appendix 1 [file publichealth_v10i1e46903_app1.zip › Figures 1-6 MA1/Figure-1/(A) Ontario.png]

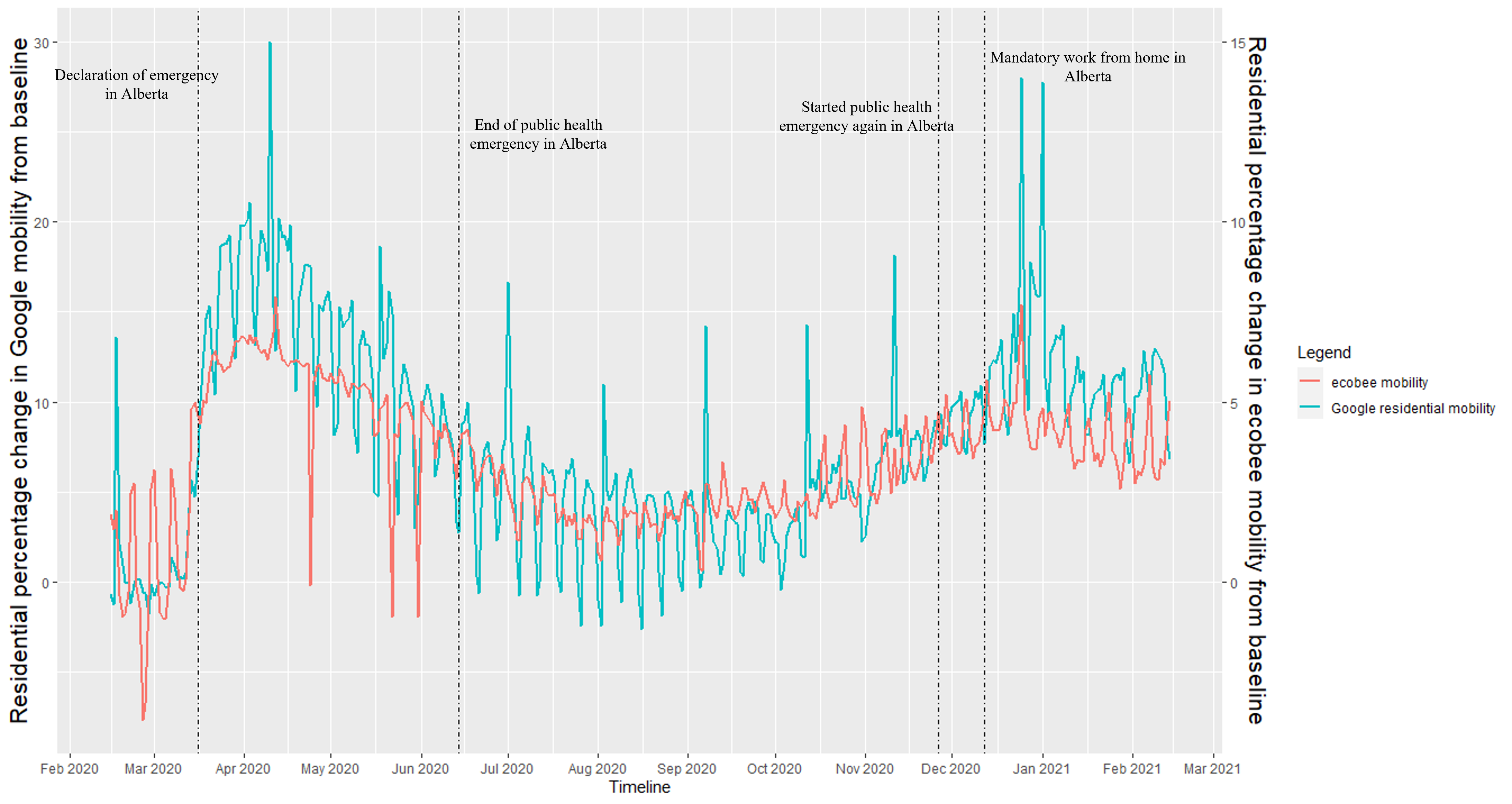

Supplement: Multimedia Appendix 1 [file publichealth_v10i1e46903_app1.zip › Figures 1-6 MA1/Figure-1/(B) Alberta.png]

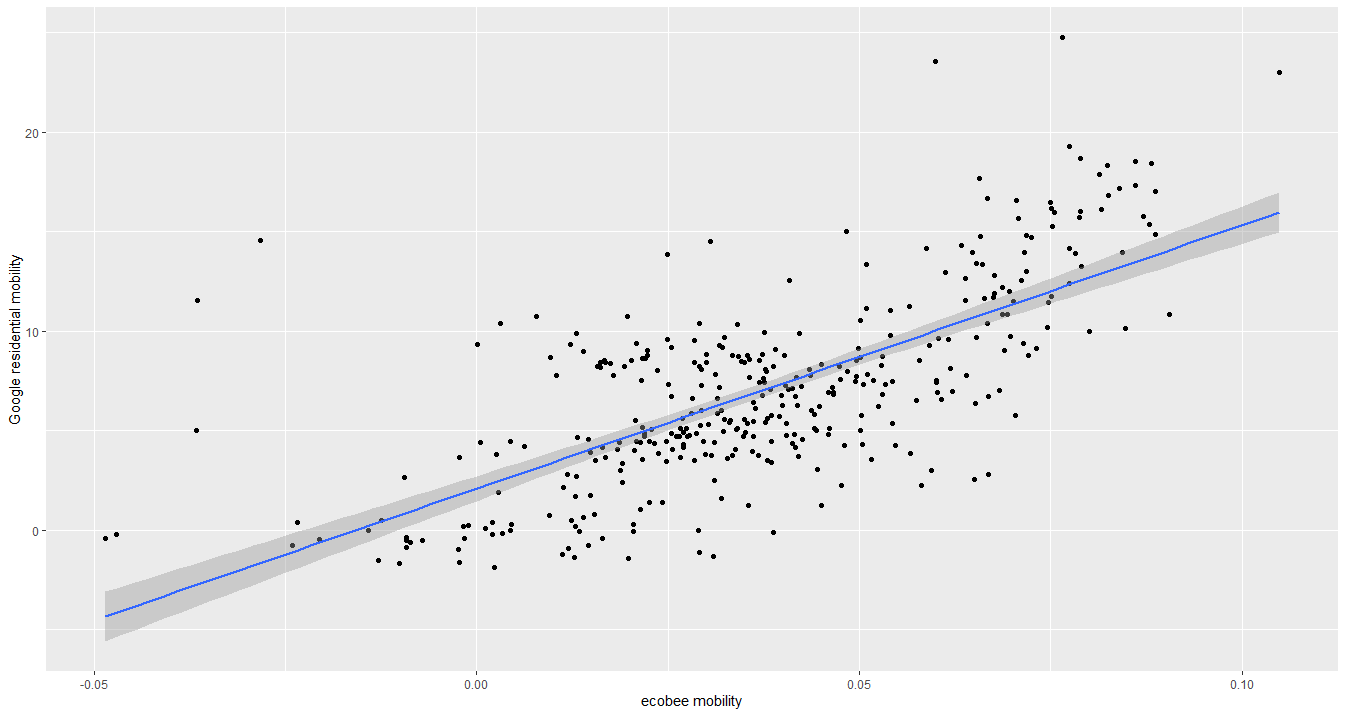

Supplement: Multimedia Appendix 1 [file publichealth_v10i1e46903_app1.zip › Figures 1-6 MA1/Figure-2/(D) British Columbia.png]

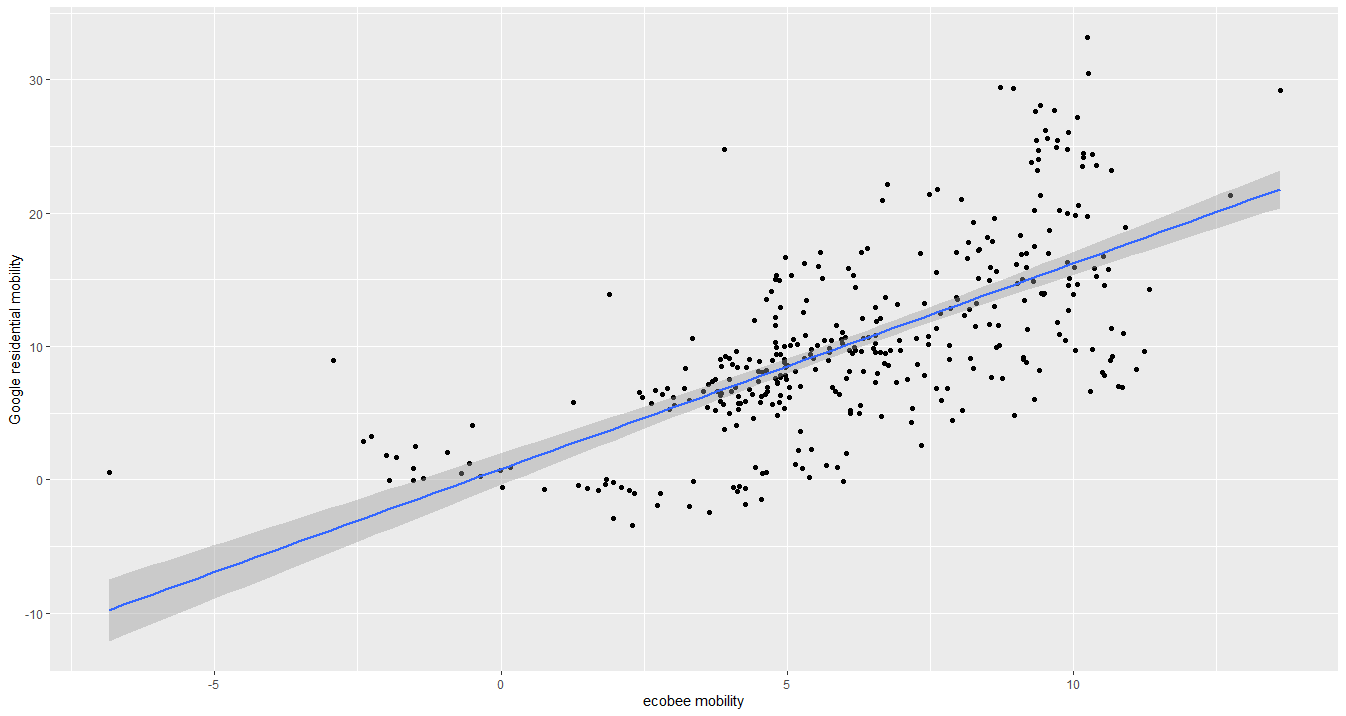

Supplement: Multimedia Appendix 1 [file publichealth_v10i1e46903_app1.zip › Figures 1-6 MA1/Figure-2/(C) Quebec.png]

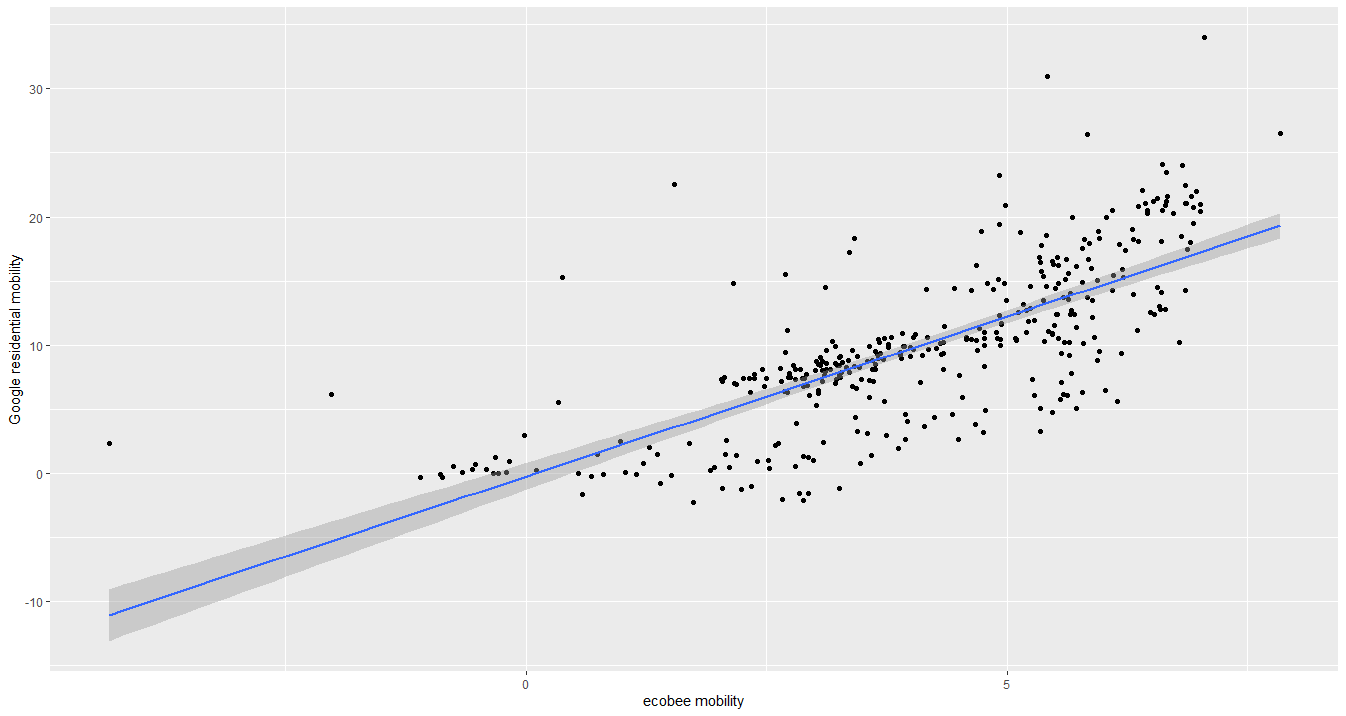

Supplement: Multimedia Appendix 1 [file publichealth_v10i1e46903_app1.zip › Figures 1-6 MA1/Figure-2/(A) Ontario.png]

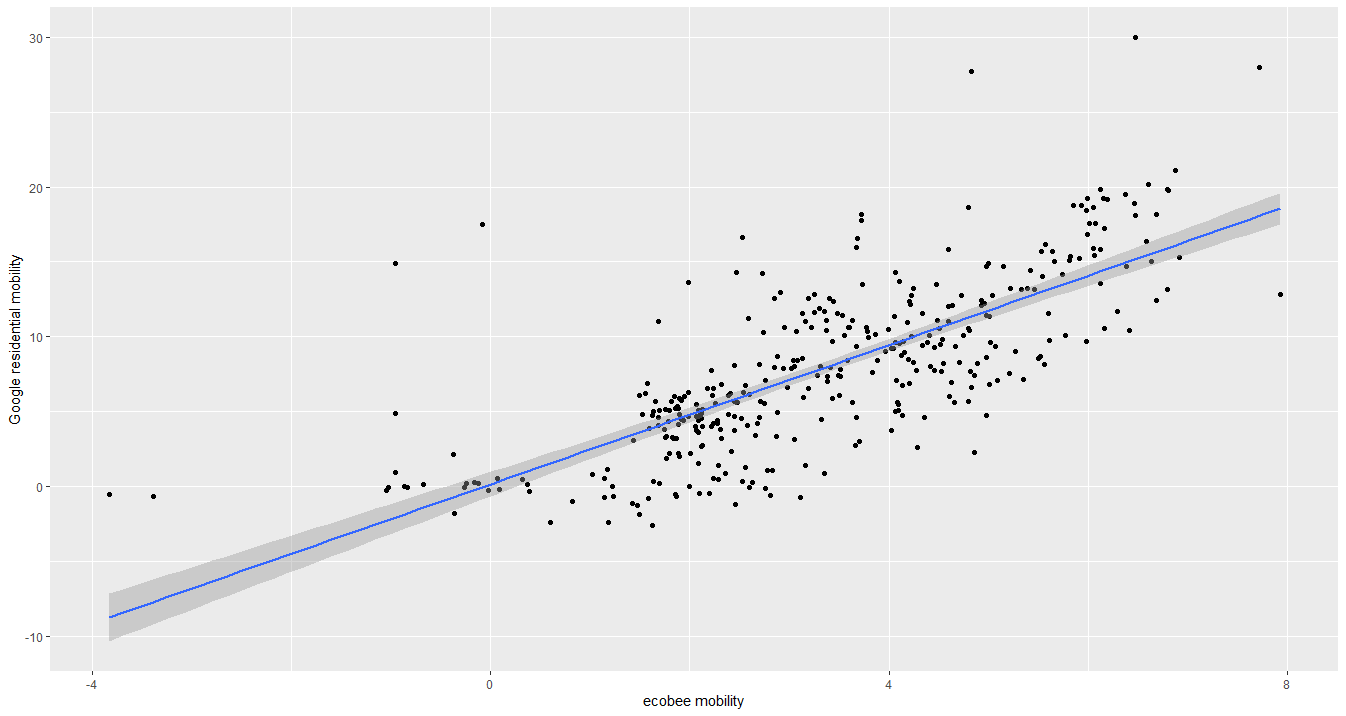

Supplement: Multimedia Appendix 1 [file publichealth_v10i1e46903_app1.zip › Figures 1-6 MA1/Figure-2/(B) Alberta.png]

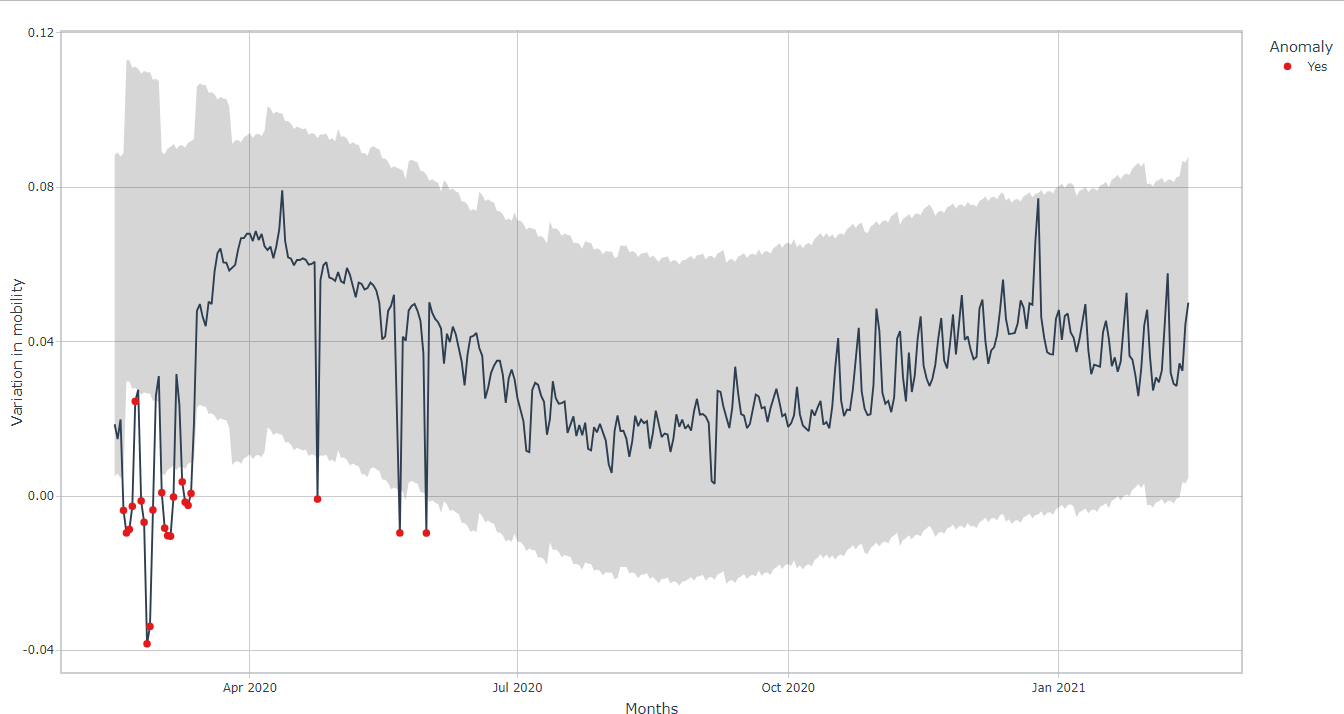

Supplement: Multimedia Appendix 1 [file publichealth_v10i1e46903_app1.zip › Figures 1-6 MA1/Figure_6/A/bc_g.png]

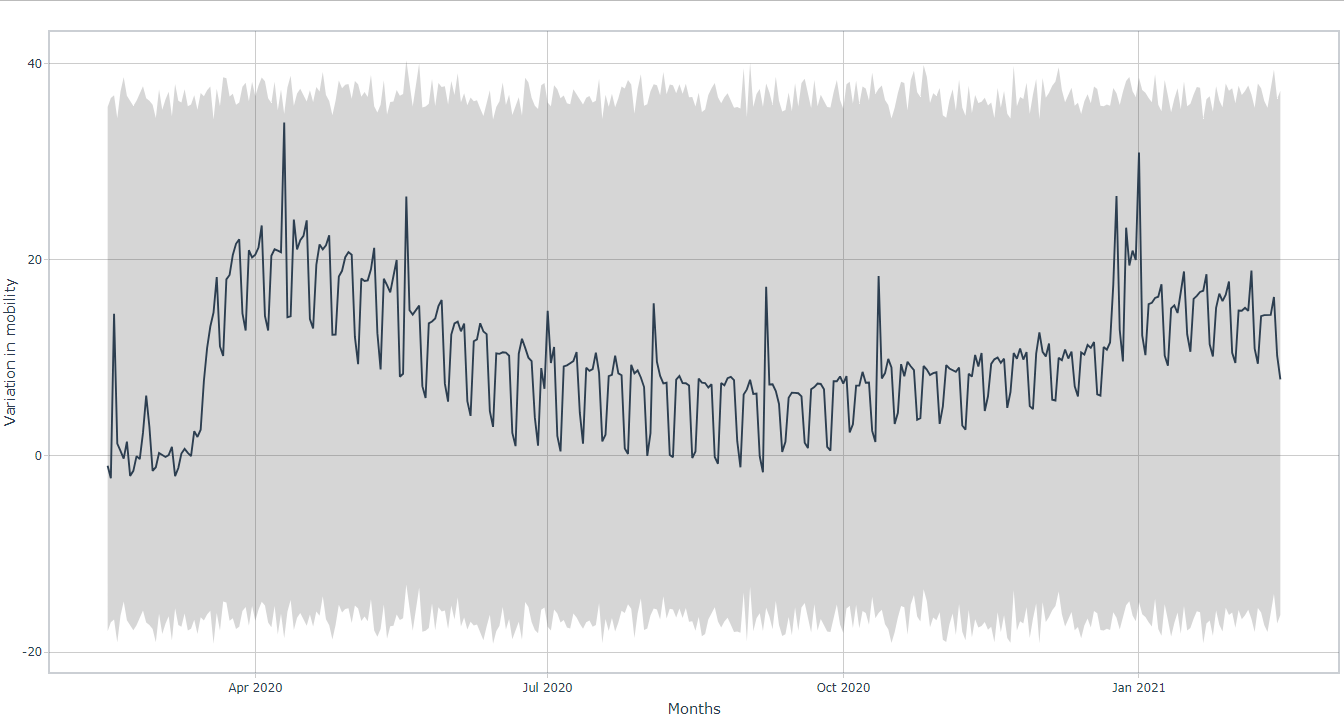

Supplement: Multimedia Appendix 1 [file publichealth_v10i1e46903_app1.zip › Figures 1-6 MA1/Figure_6/A/on_g.png]

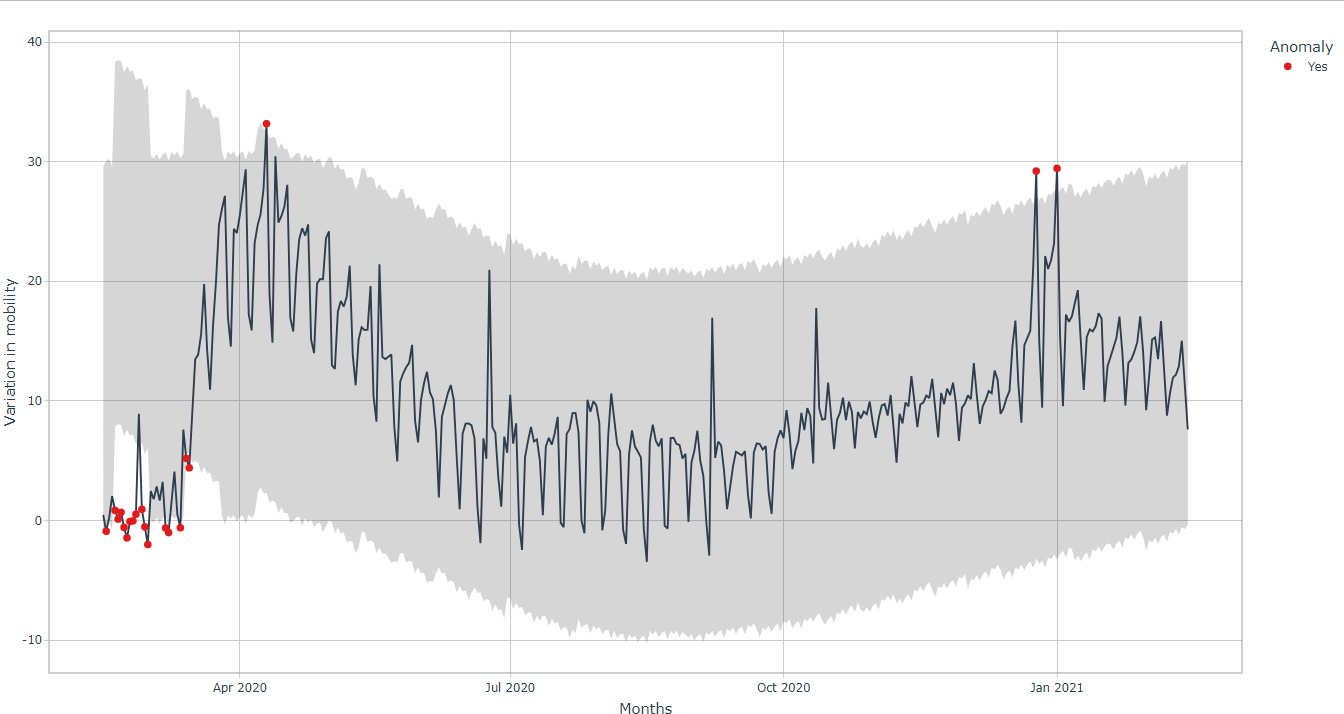

Supplement: Multimedia Appendix 1 [file publichealth_v10i1e46903_app1.zip › Figures 1-6 MA1/Figure_6/A/qc_g.png]

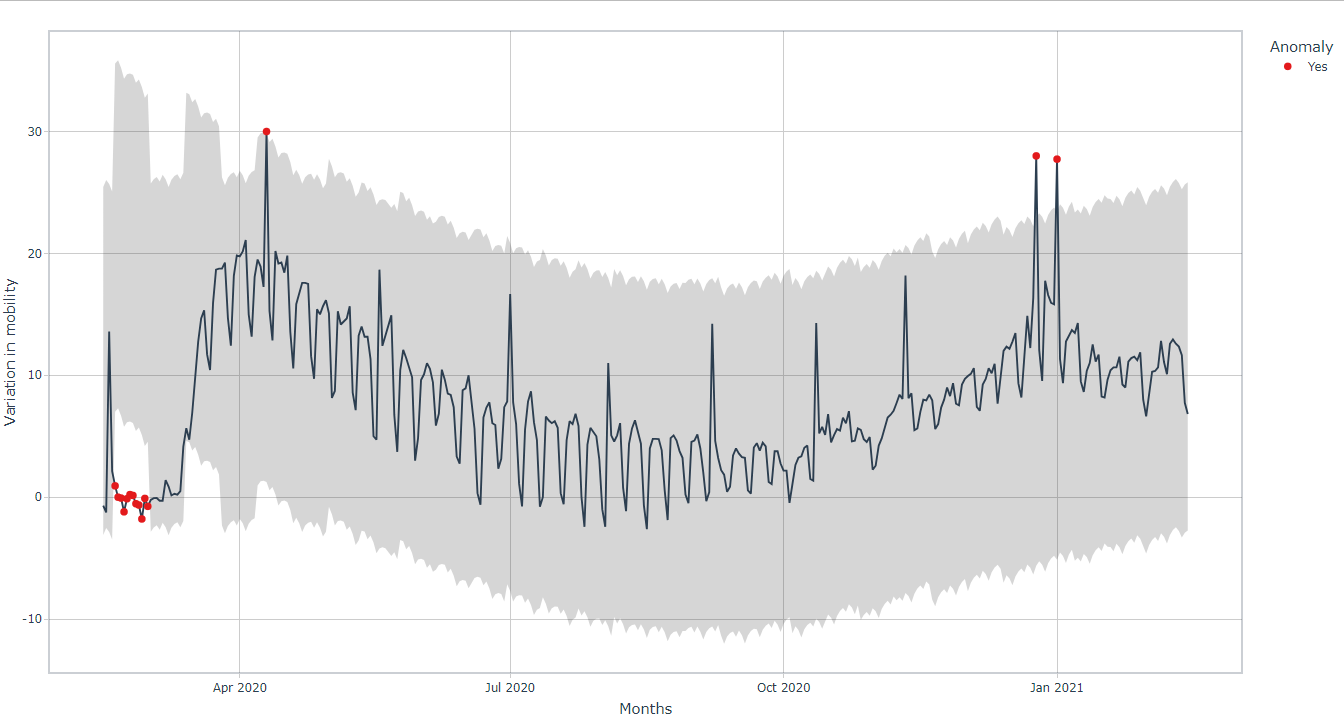

Supplement: Multimedia Appendix 1 [file publichealth_v10i1e46903_app1.zip › Figures 1-6 MA1/Figure_6/A/ab_g.png]

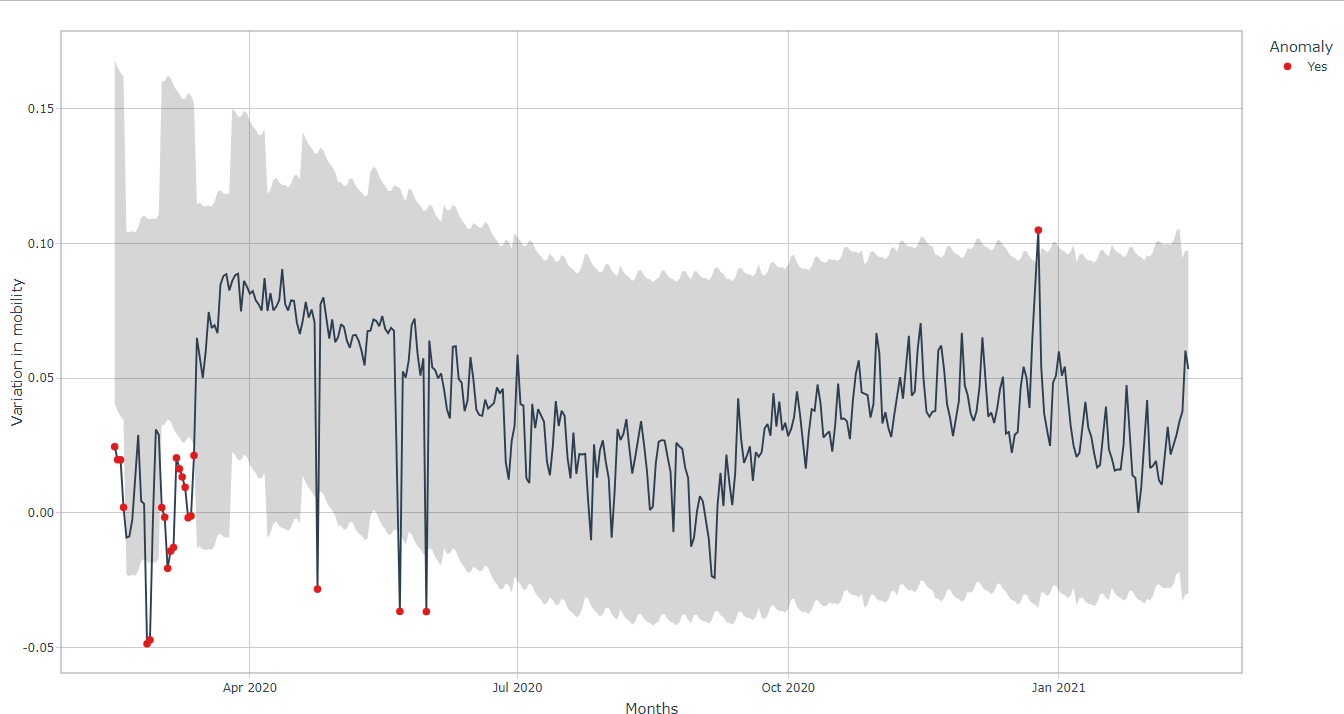

Supplement: Multimedia Appendix 1 [file publichealth_v10i1e46903_app1.zip › Figures 1-6 MA1/Figure_6/B/bc_e.png]

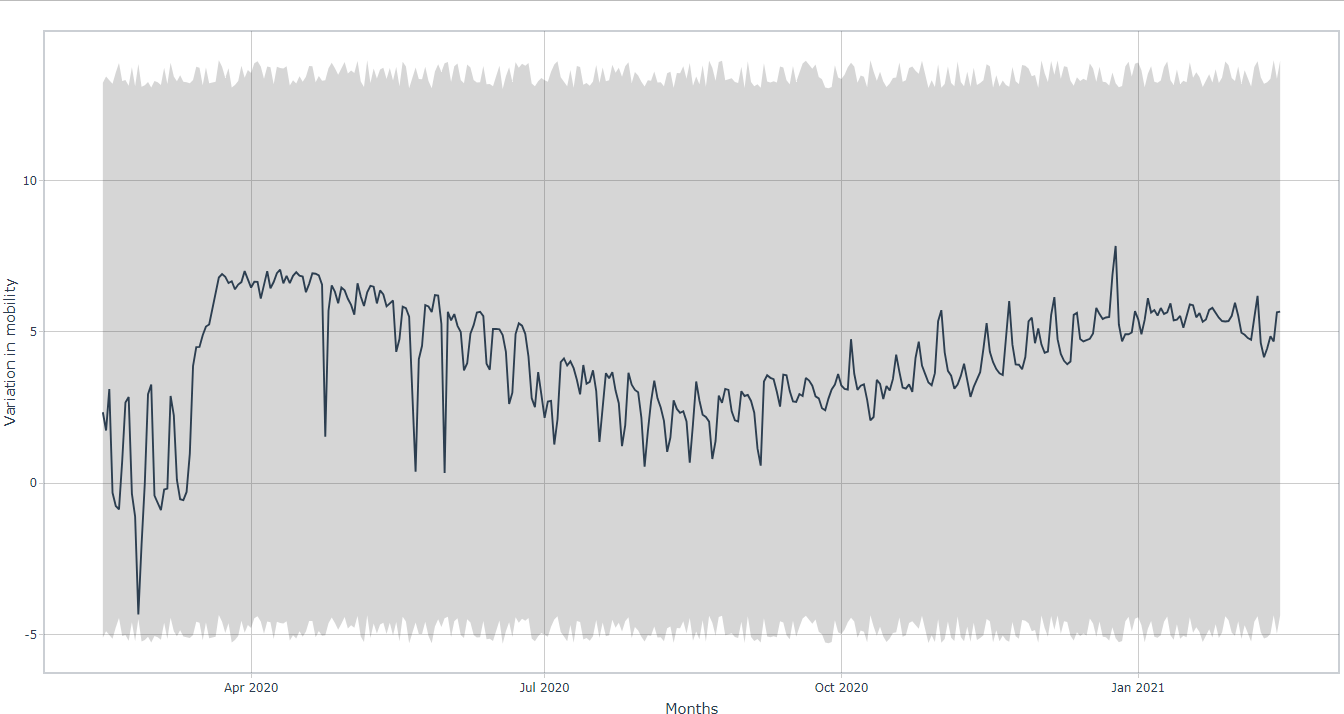

Supplement: Multimedia Appendix 1 [file publichealth_v10i1e46903_app1.zip › Figures 1-6 MA1/Figure_6/B/on_e.png]

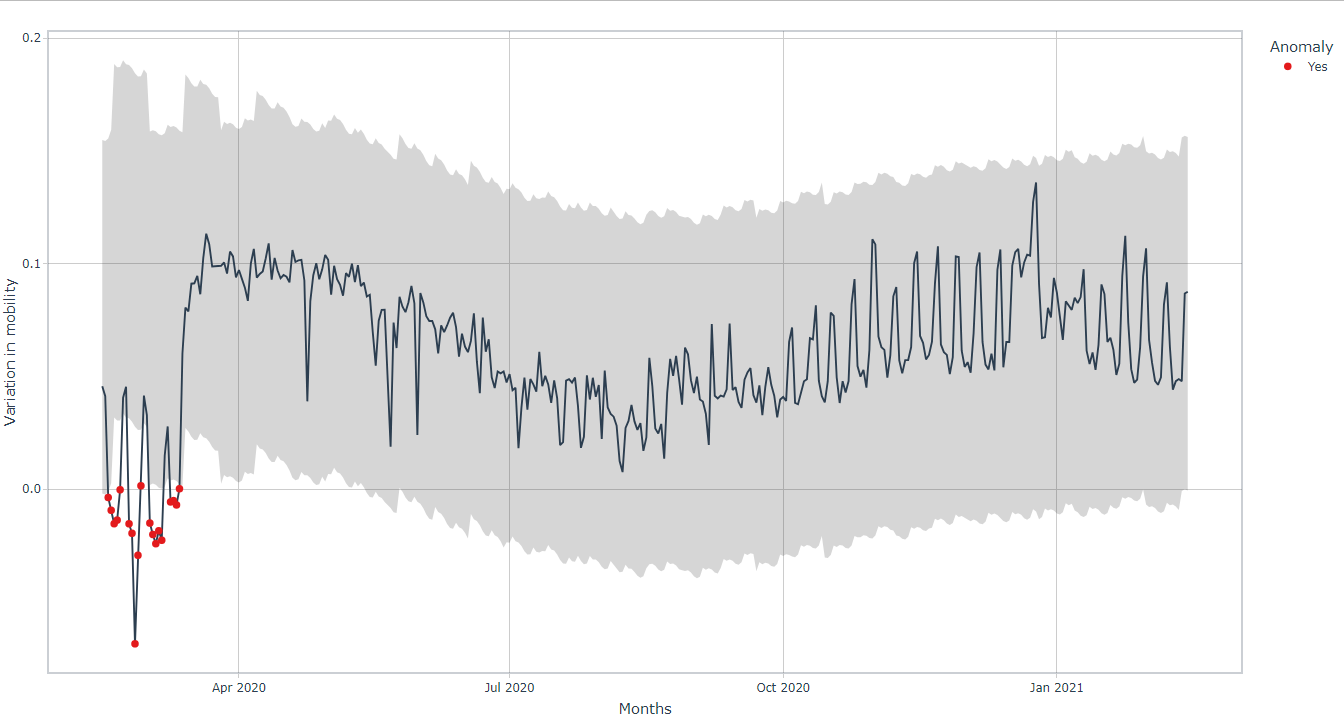

Supplement: Multimedia Appendix 1 [file publichealth_v10i1e46903_app1.zip › Figures 1-6 MA1/Figure_6/B/qc_e.png]

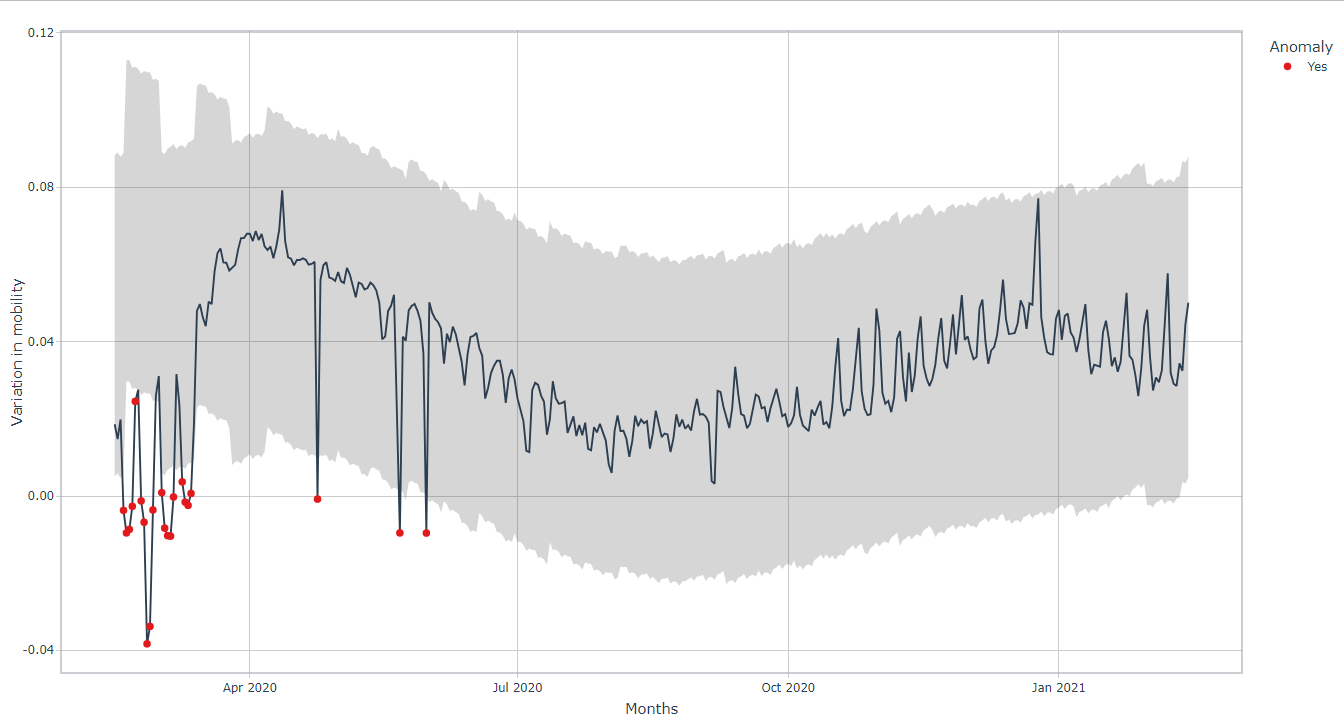

Supplement: Multimedia Appendix 1 [file publichealth_v10i1e46903_app1.zip › Figures 1-6 MA1/Figure_6/B/ab_e.png]

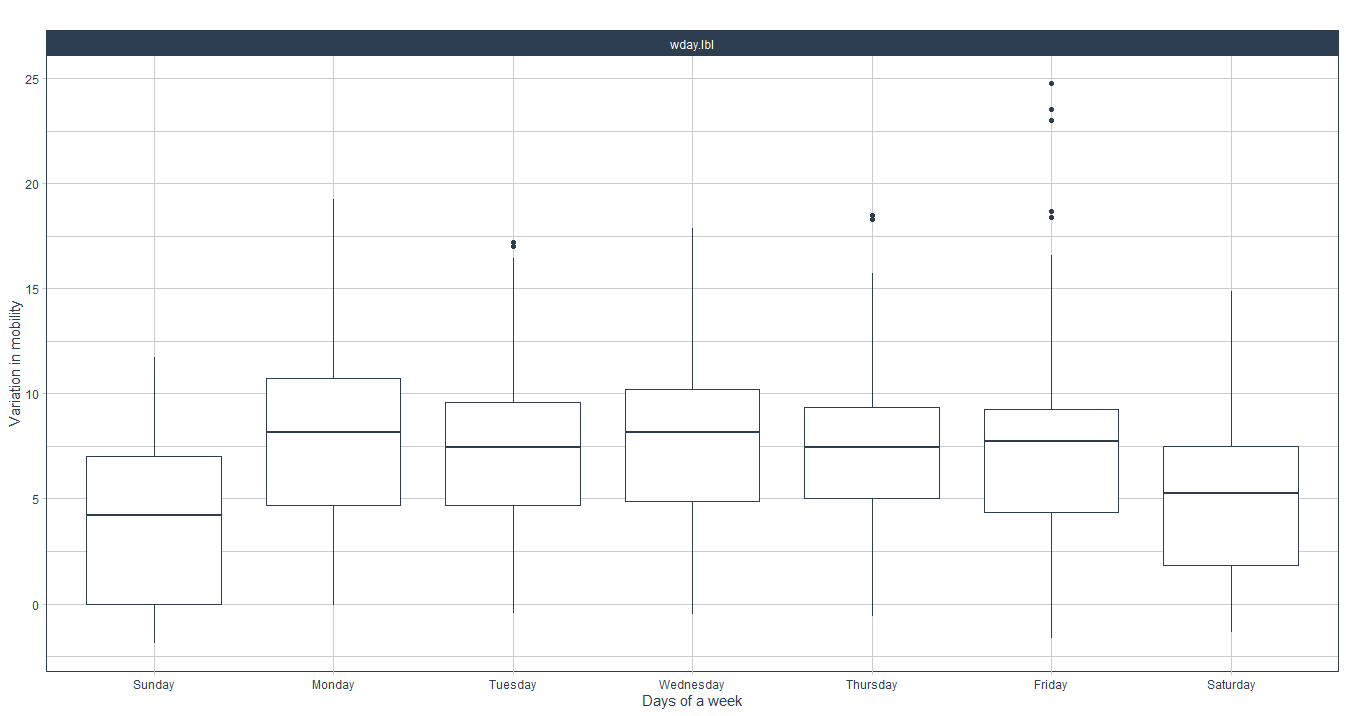

Supplement: Multimedia Appendix 1 [file publichealth_v10i1e46903_app1.zip › Figures 1-6 MA1/Figure-3/A/bc_g.png]

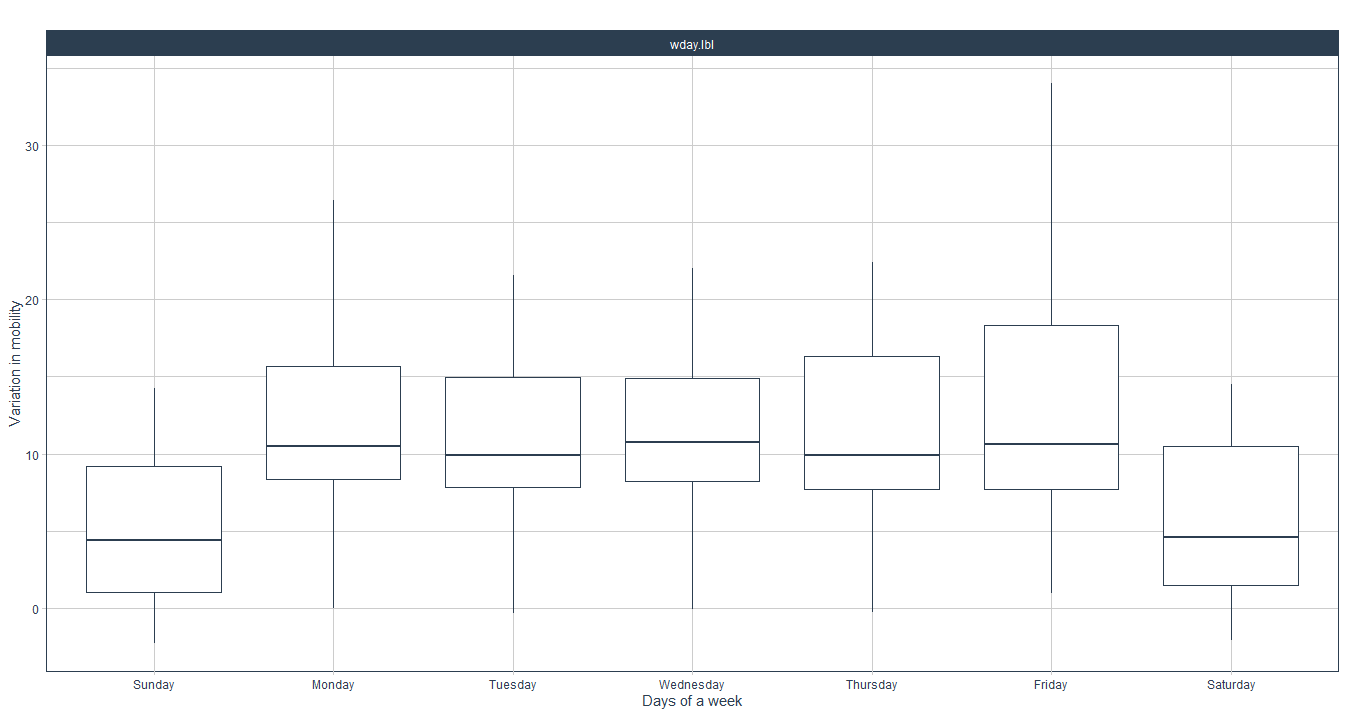

Supplement: Multimedia Appendix 1 [file publichealth_v10i1e46903_app1.zip › Figures 1-6 MA1/Figure-3/A/on_g.png]

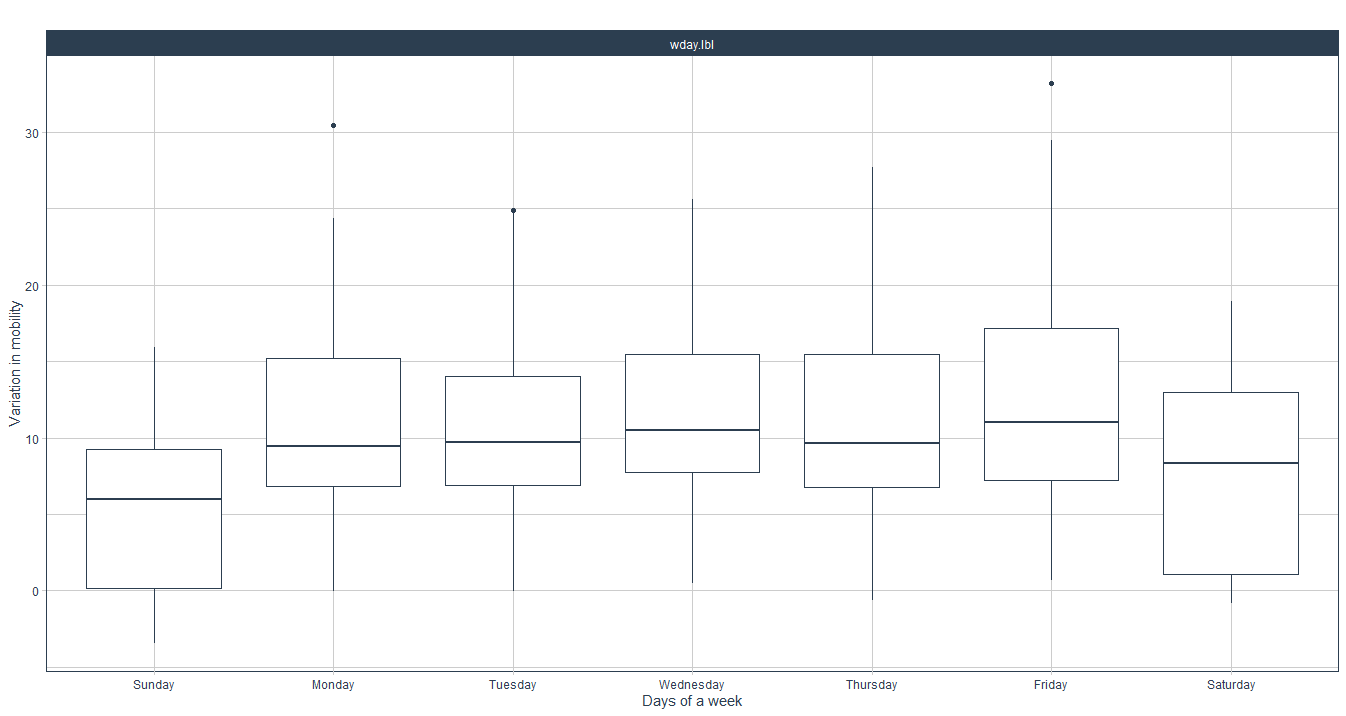

Supplement: Multimedia Appendix 1 [file publichealth_v10i1e46903_app1.zip › Figures 1-6 MA1/Figure-3/A/qc_g.png]

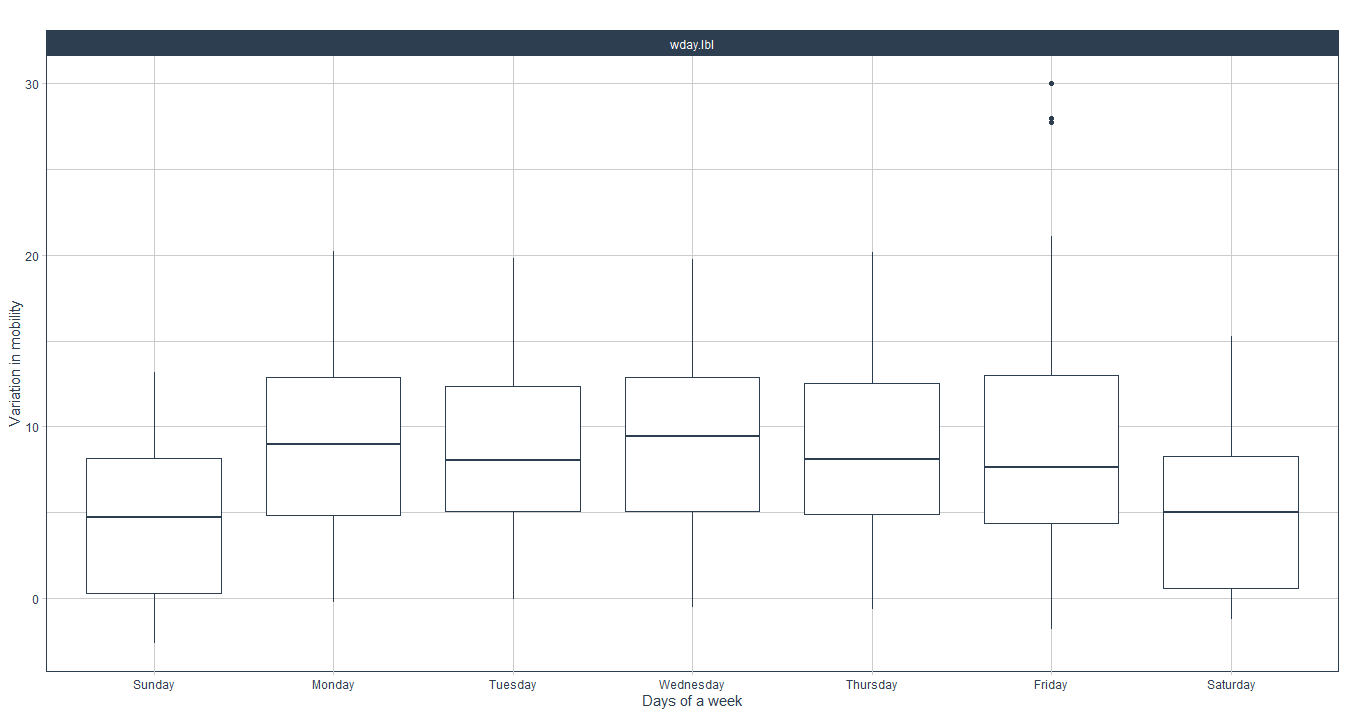

Supplement: Multimedia Appendix 1 [file publichealth_v10i1e46903_app1.zip › Figures 1-6 MA1/Figure-3/A/ab_g.png]

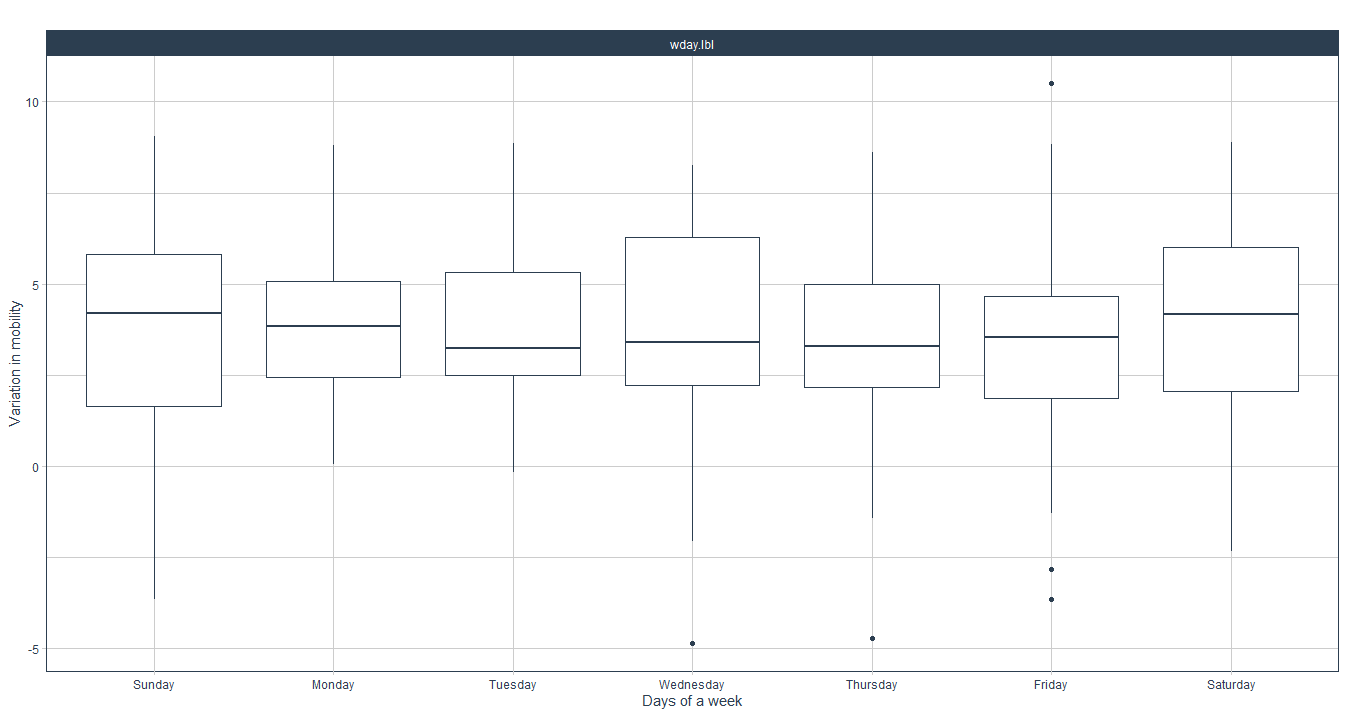

Supplement: Multimedia Appendix 1 [file publichealth_v10i1e46903_app1.zip › Figures 1-6 MA1/Figure-3/B/bc_e.png]

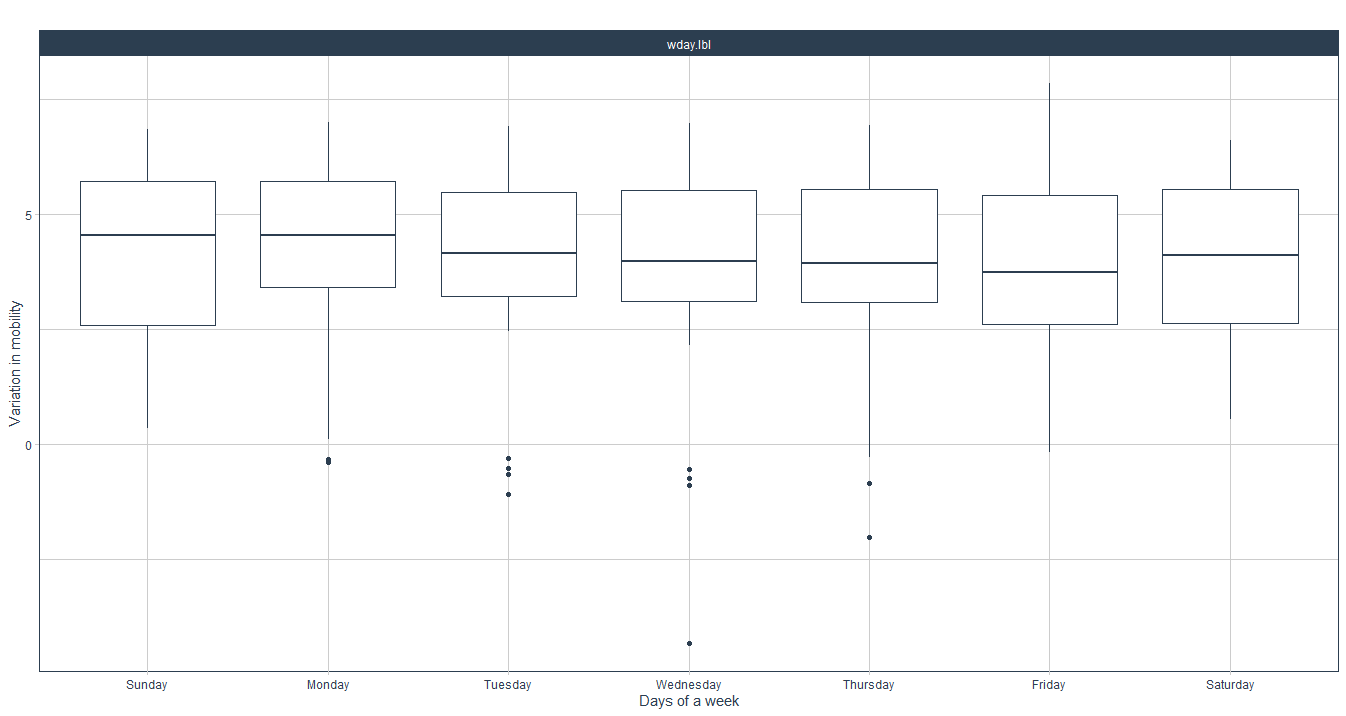

Supplement: Multimedia Appendix 1 [file publichealth_v10i1e46903_app1.zip › Figures 1-6 MA1/Figure-3/B/on_e.png]

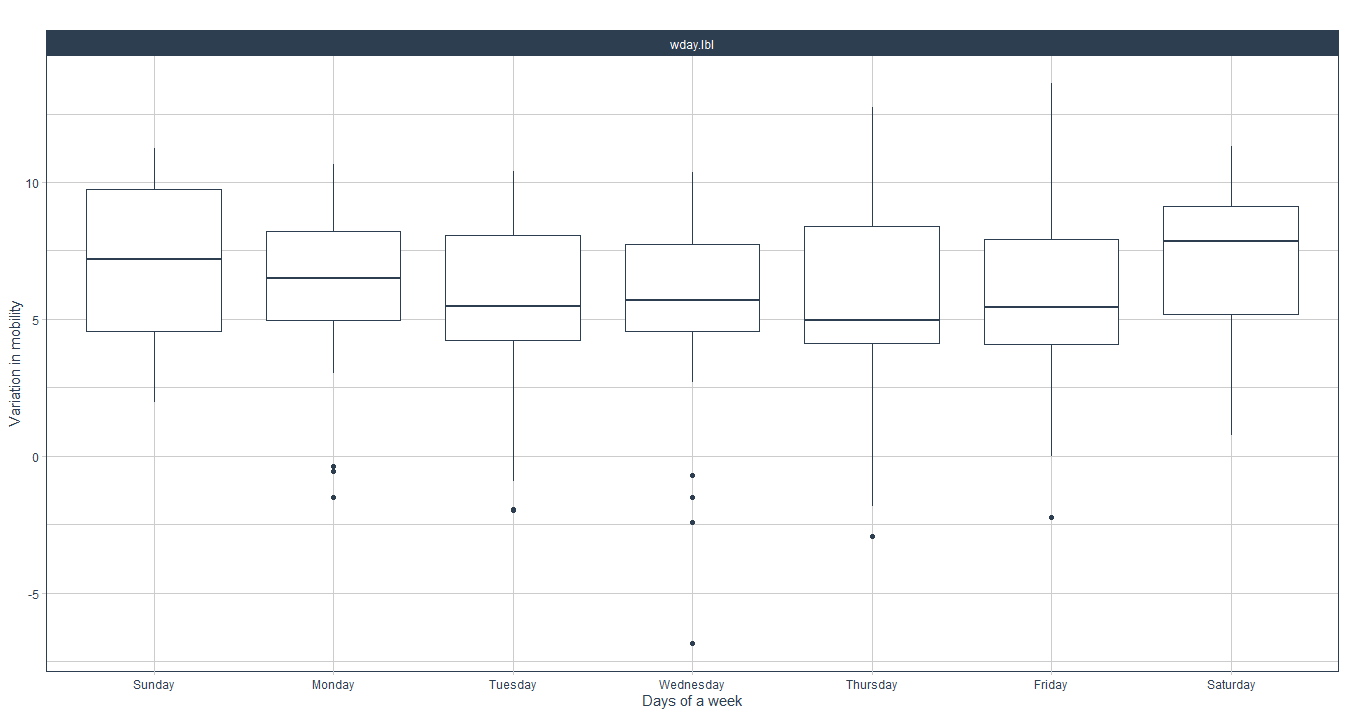

Supplement: Multimedia Appendix 1 [file publichealth_v10i1e46903_app1.zip › Figures 1-6 MA1/Figure-3/B/qc_e.png]

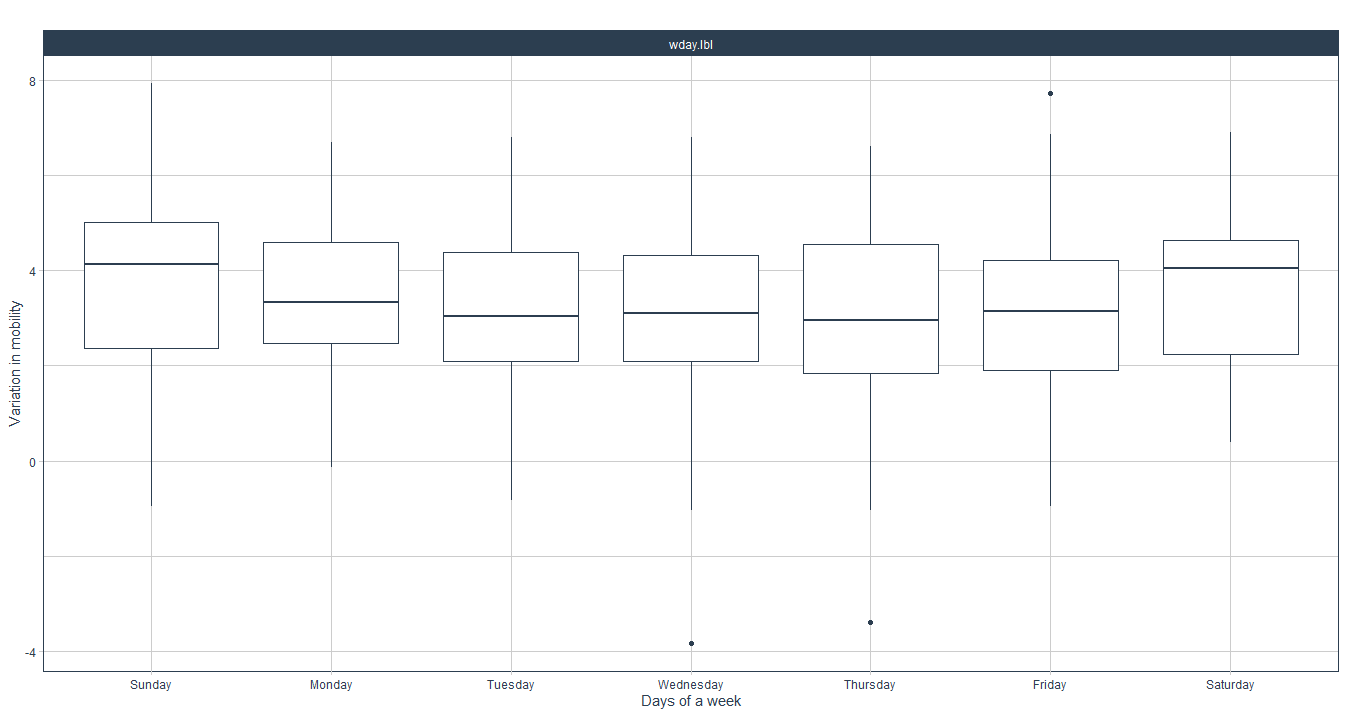

Supplement: Multimedia Appendix 1 [file publichealth_v10i1e46903_app1.zip › Figures 1-6 MA1/Figure-3/B/ab_e.png]

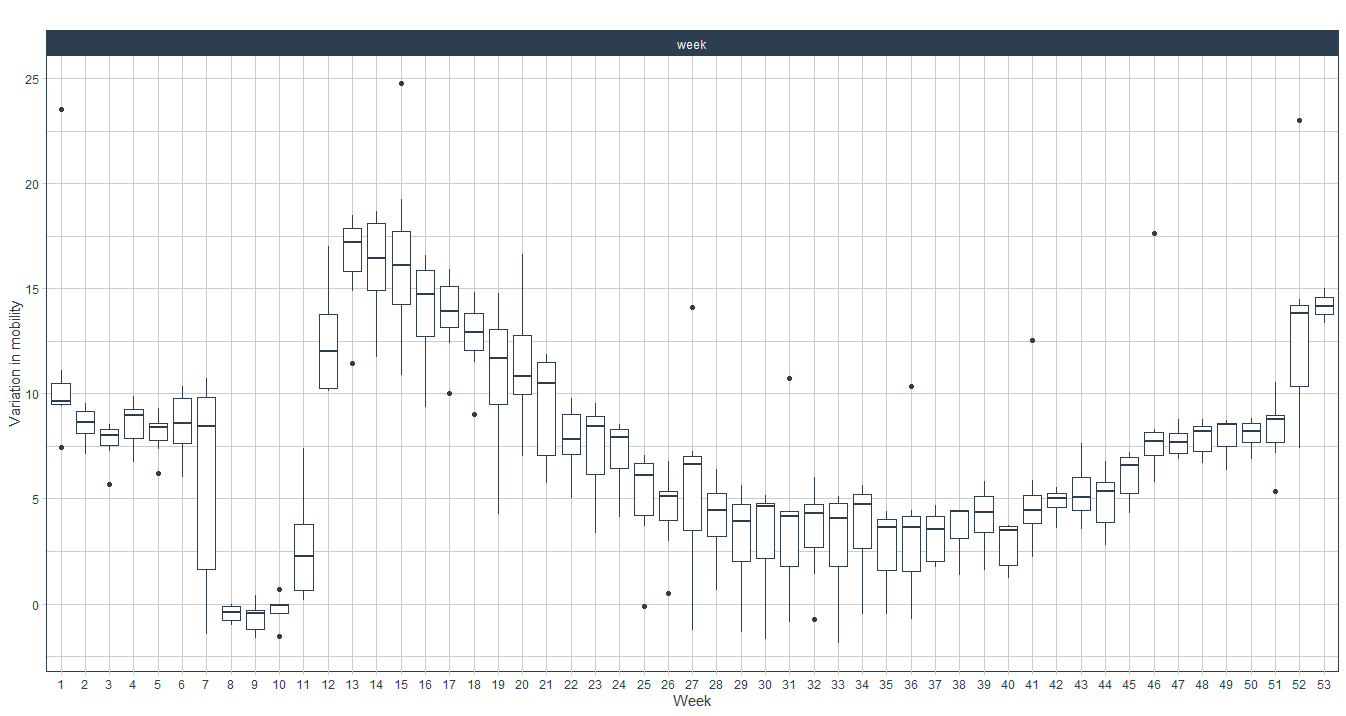

Supplement: Multimedia Appendix 1 [file publichealth_v10i1e46903_app1.zip › Figures 1-6 MA1/Figure-5/A/bc_g.png]

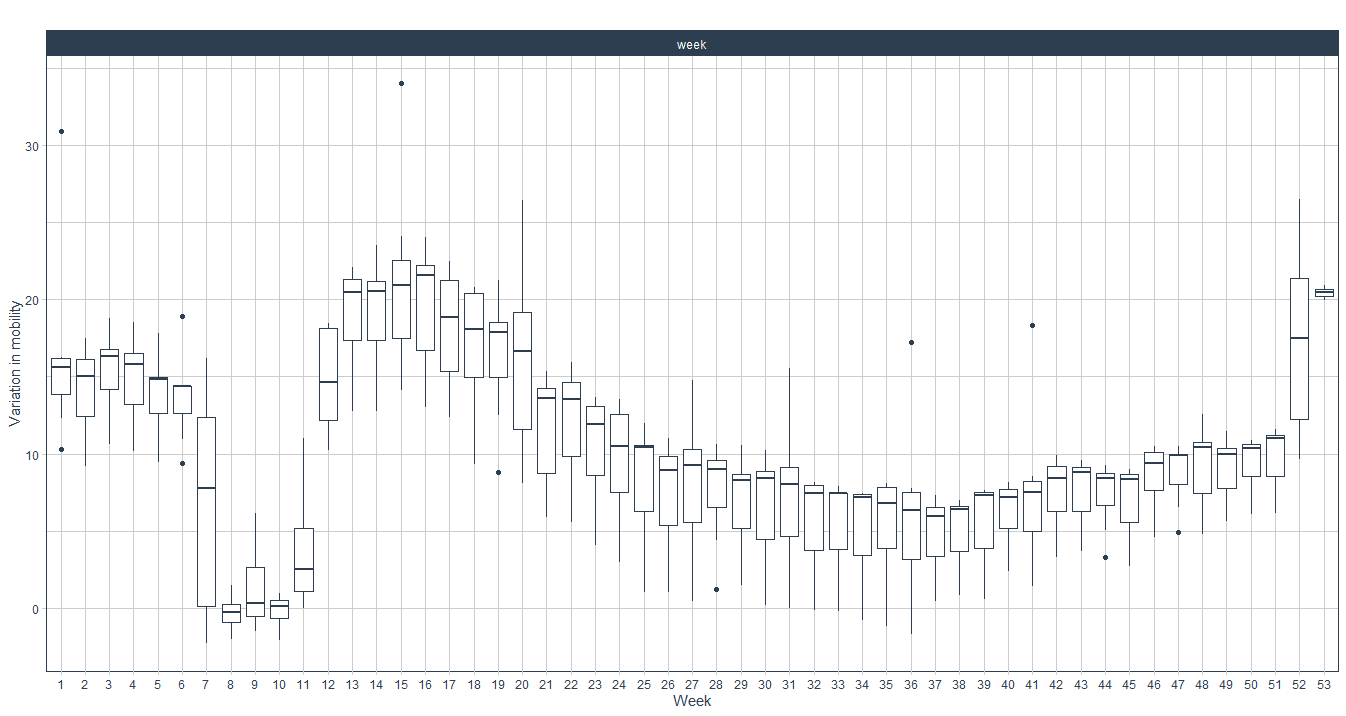

Supplement: Multimedia Appendix 1 [file publichealth_v10i1e46903_app1.zip › Figures 1-6 MA1/Figure-5/A/on_g.png]

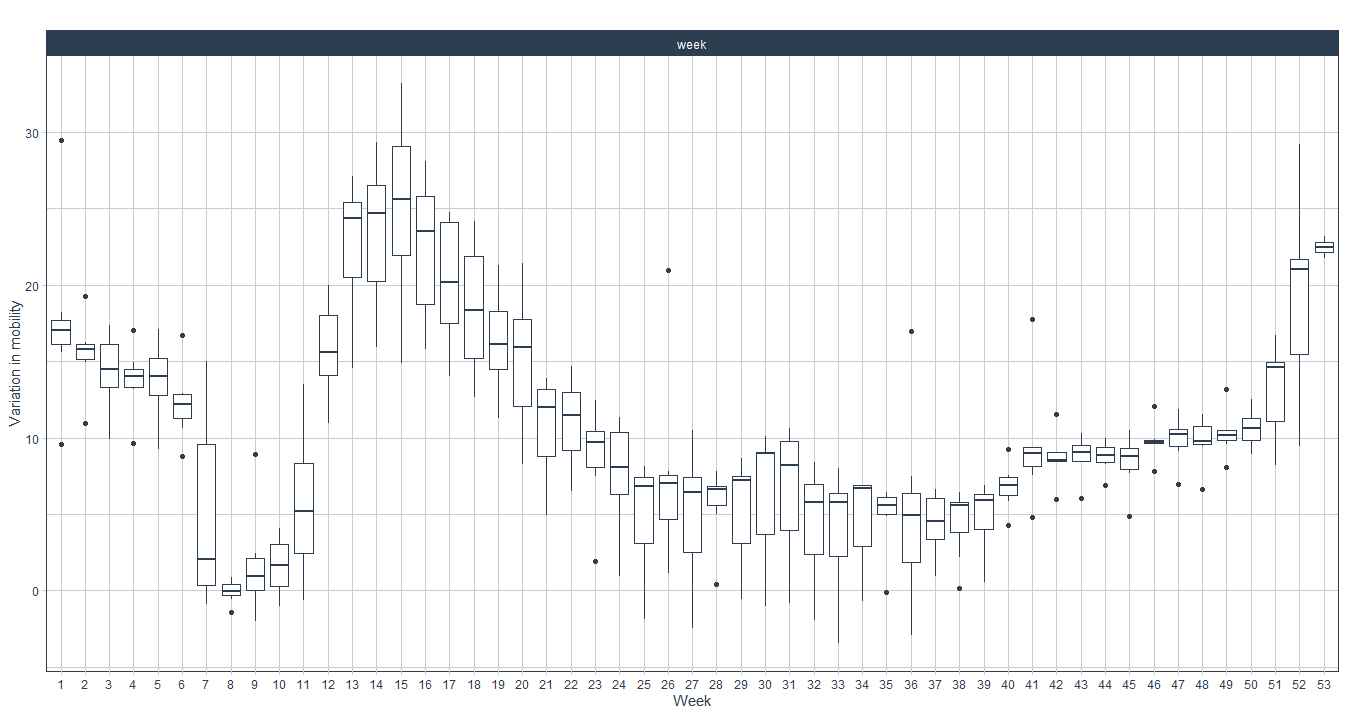

Supplement: Multimedia Appendix 1 [file publichealth_v10i1e46903_app1.zip › Figures 1-6 MA1/Figure-5/A/qc_g.png]

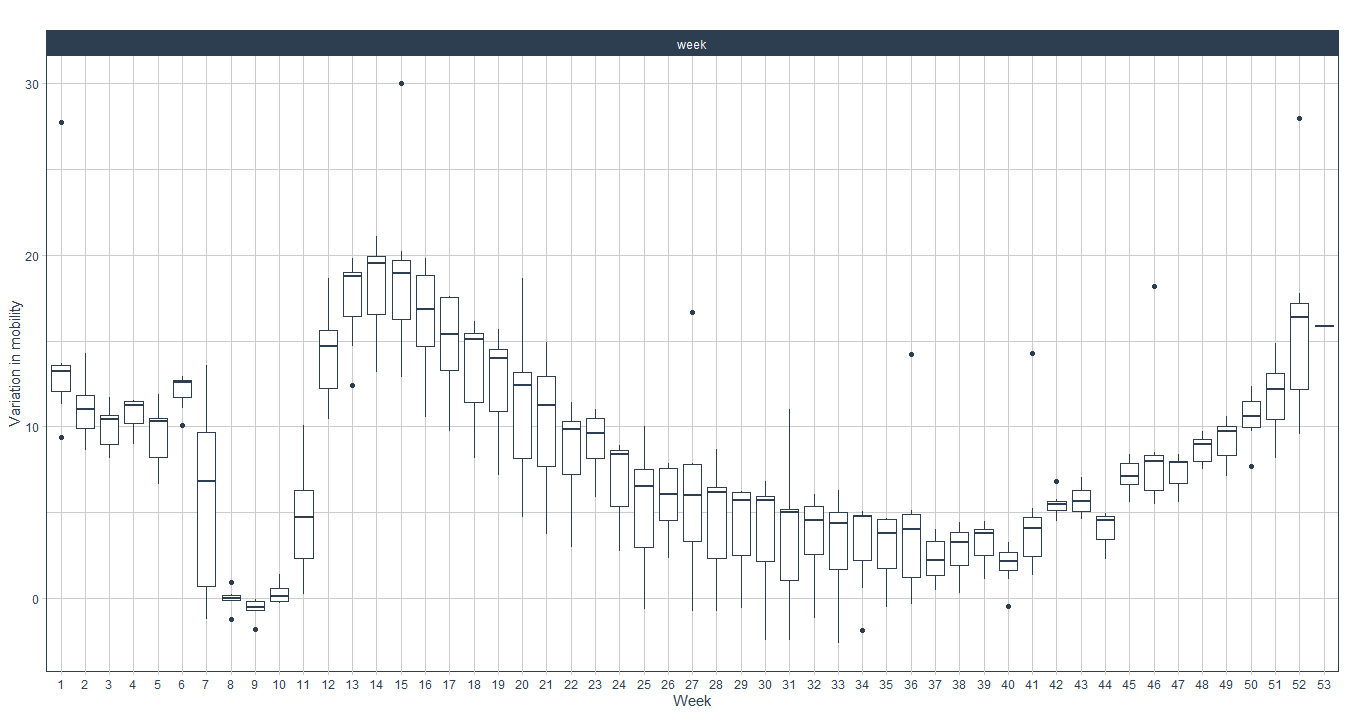

Supplement: Multimedia Appendix 1 [file publichealth_v10i1e46903_app1.zip › Figures 1-6 MA1/Figure-5/A/ab_g.png]

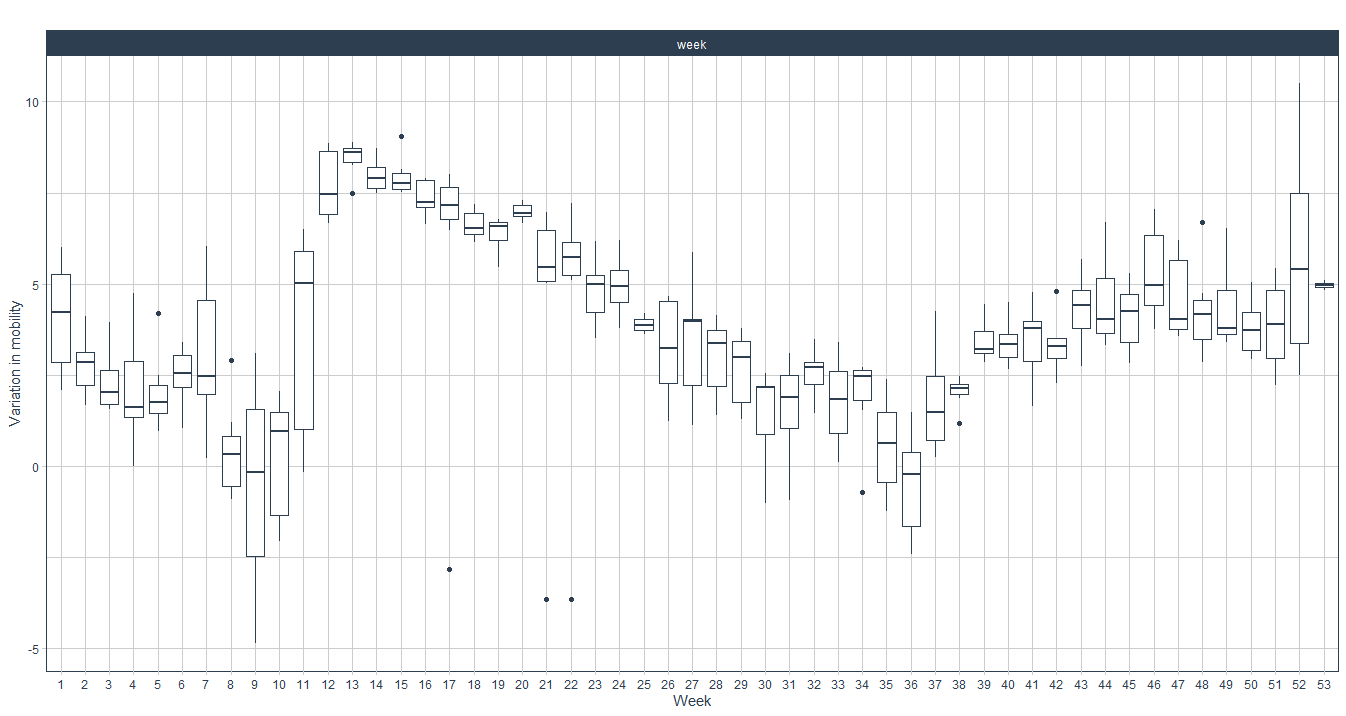

Supplement: Multimedia Appendix 1 [file publichealth_v10i1e46903_app1.zip › Figures 1-6 MA1/Figure-5/B/bc_e.png]

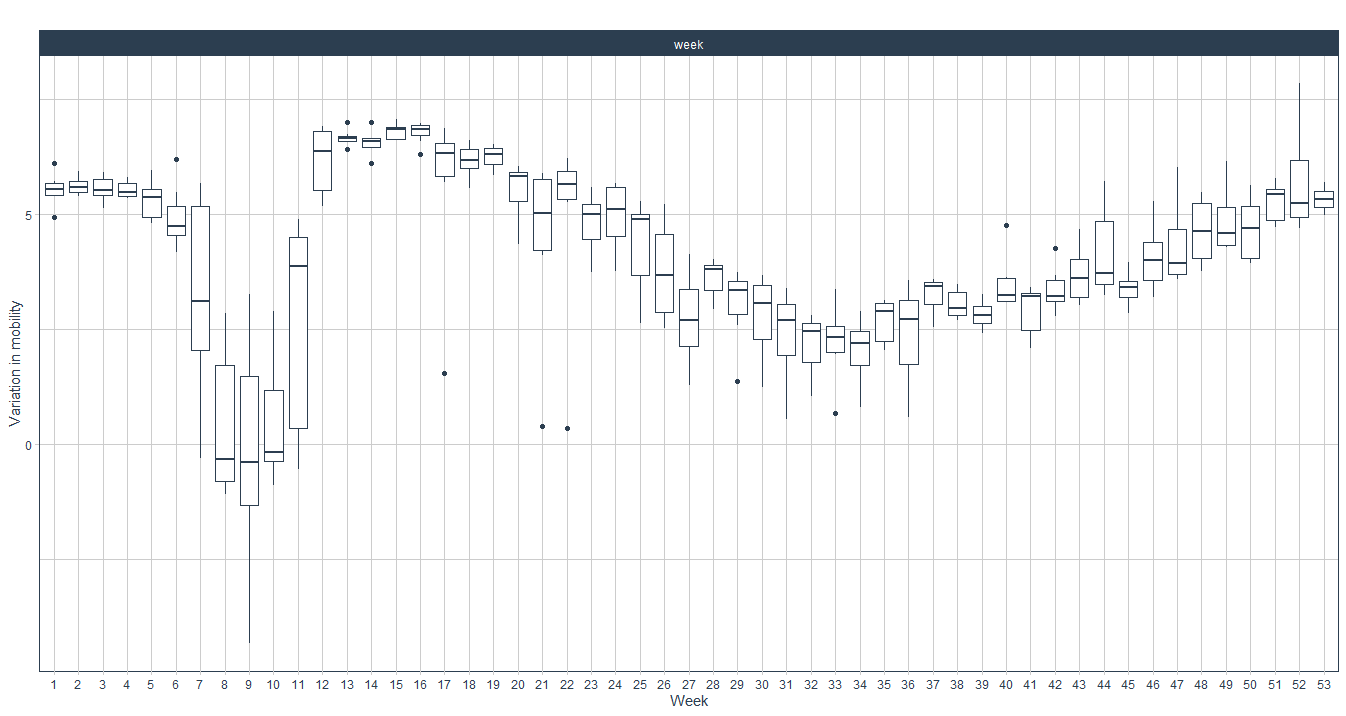

Supplement: Multimedia Appendix 1 [file publichealth_v10i1e46903_app1.zip › Figures 1-6 MA1/Figure-5/B/on_e.png]

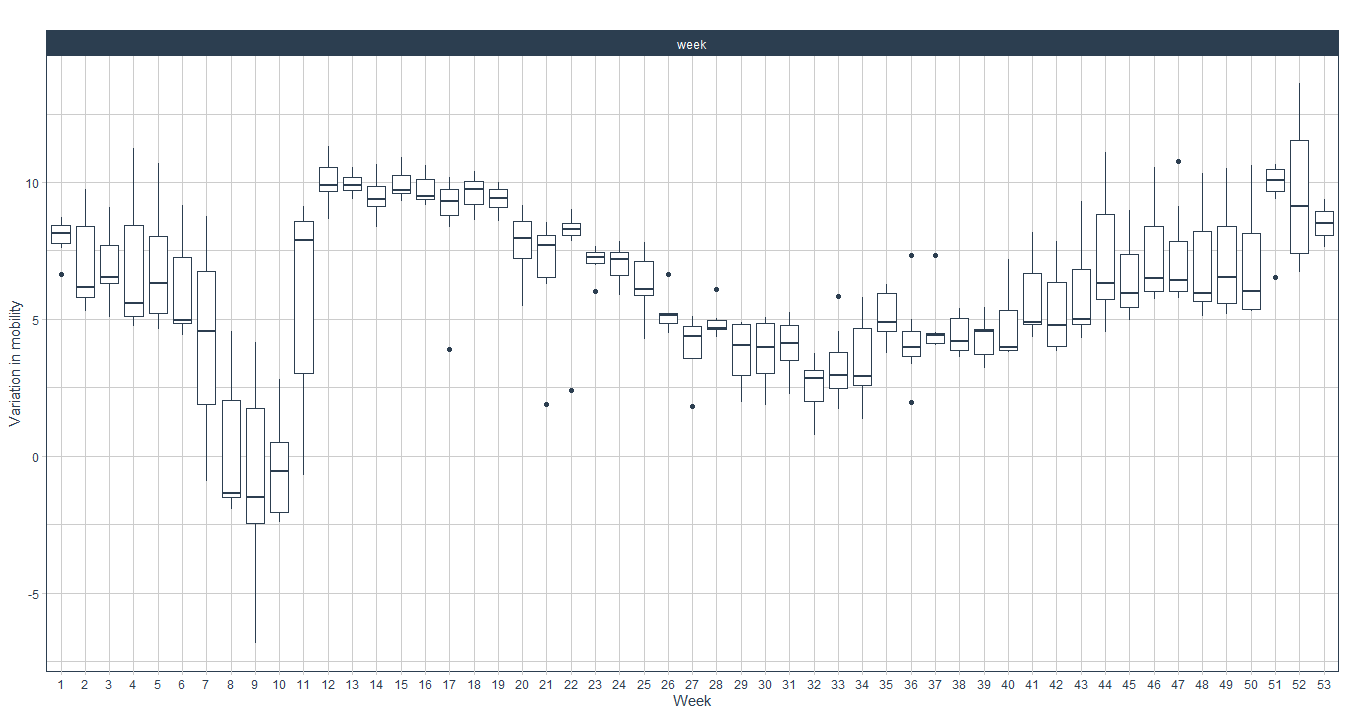

Supplement: Multimedia Appendix 1 [file publichealth_v10i1e46903_app1.zip › Figures 1-6 MA1/Figure-5/B/qc_e.png]

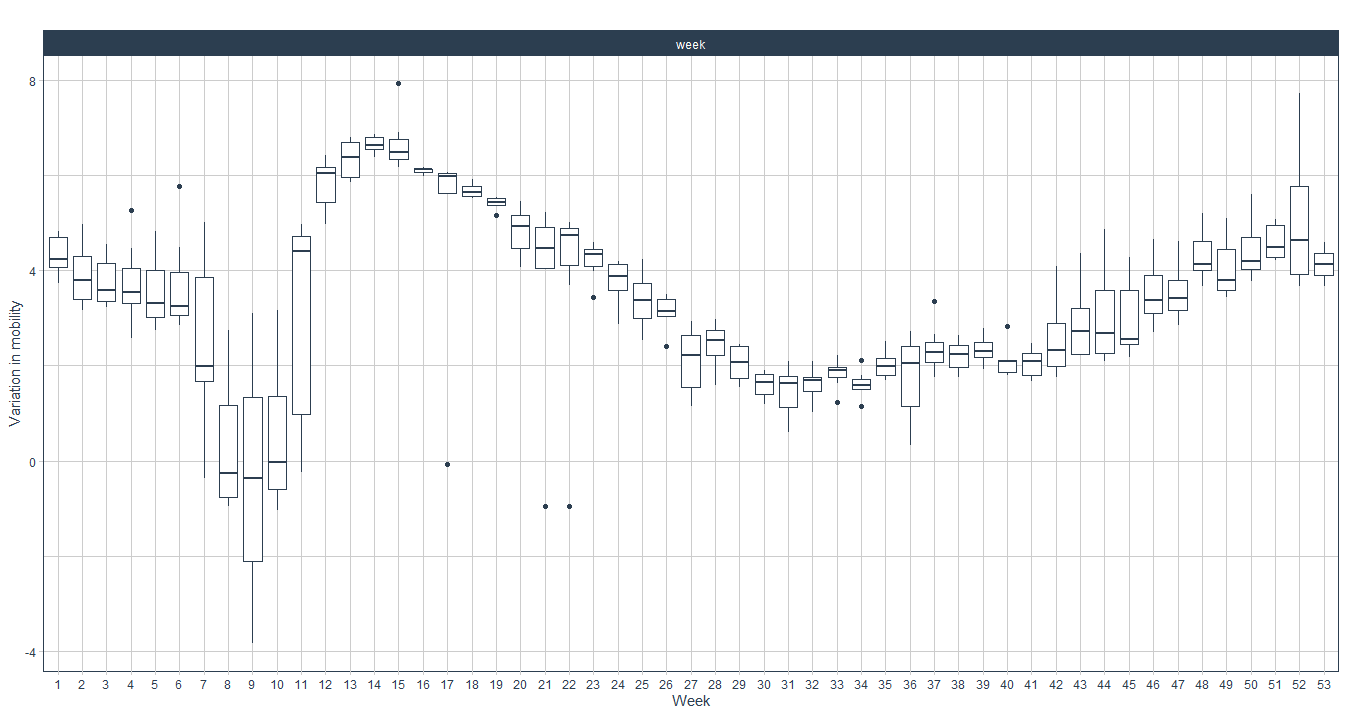

Supplement: Multimedia Appendix 1 [file publichealth_v10i1e46903_app1.zip › Figures 1-6 MA1/Figure-5/B/ab_e.png]

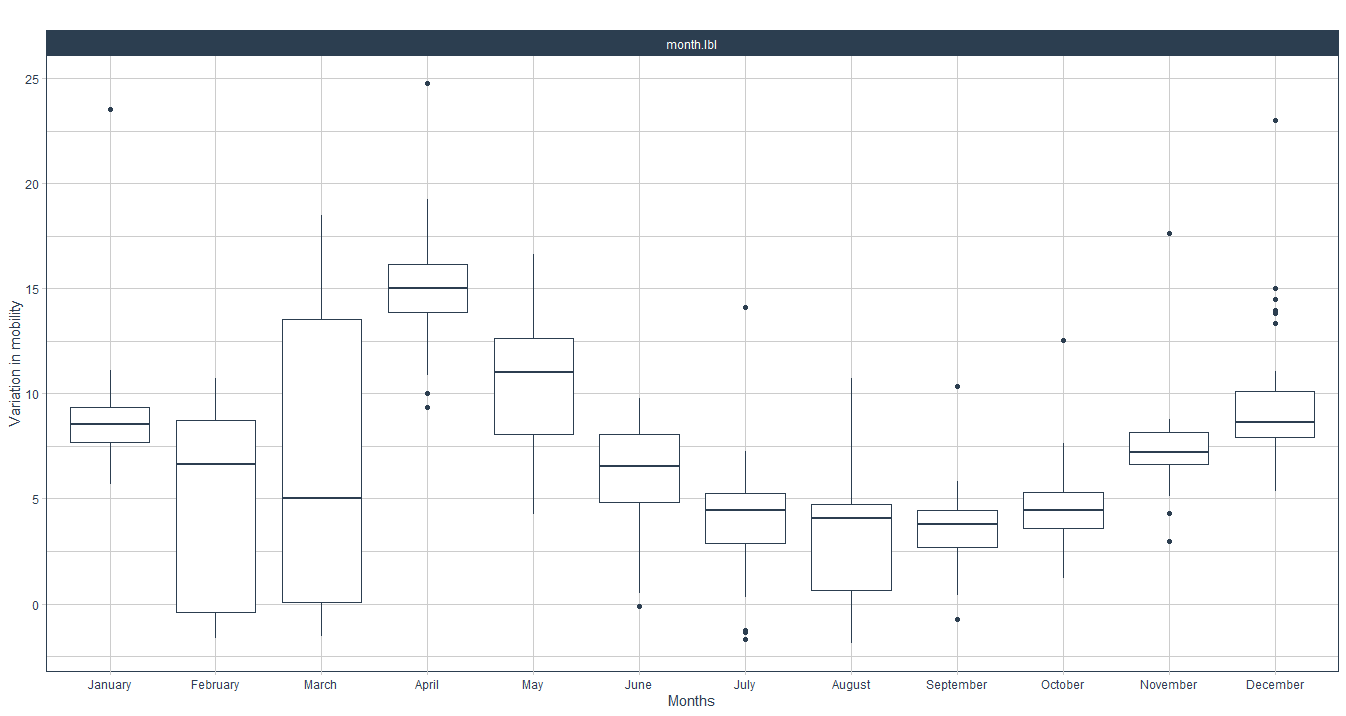

Supplement: Multimedia Appendix 1 [file publichealth_v10i1e46903_app1.zip › Figures 1-6 MA1/Figure_4/A/bc_g.png]

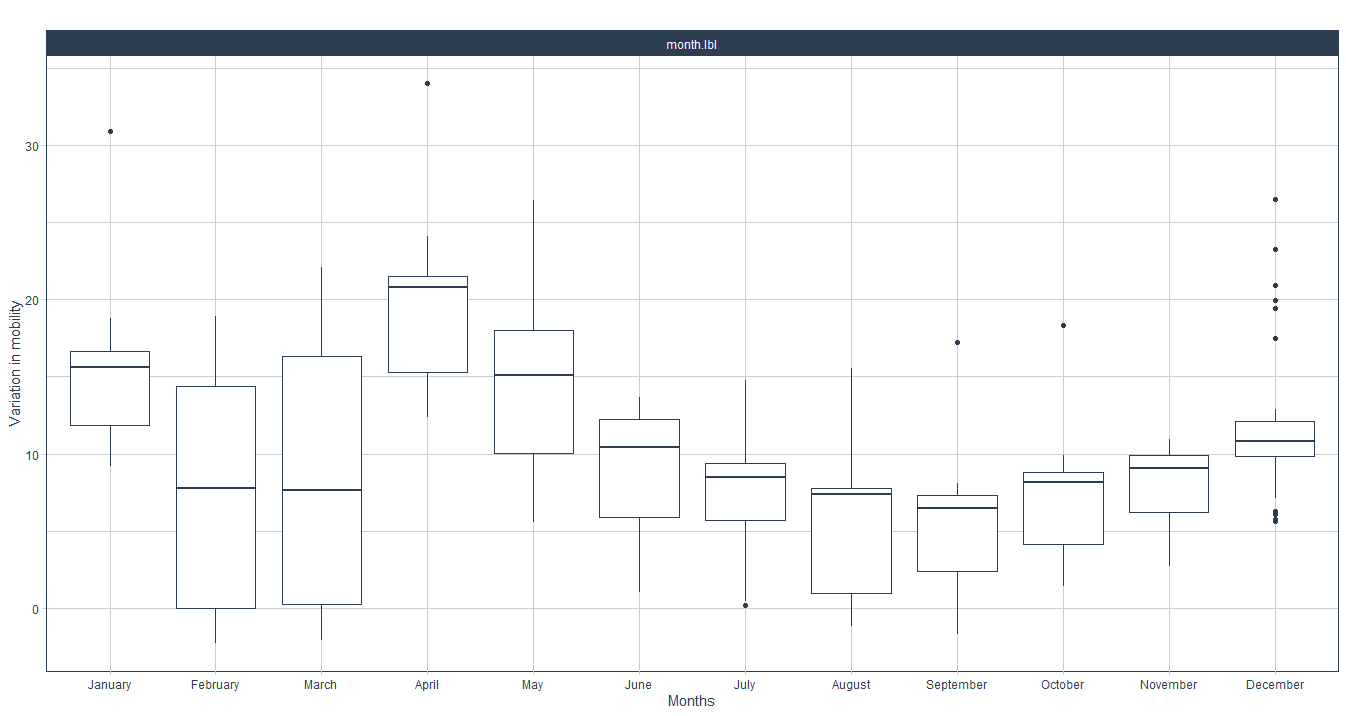

Supplement: Multimedia Appendix 1 [file publichealth_v10i1e46903_app1.zip › Figures 1-6 MA1/Figure_4/A/on_g.png]

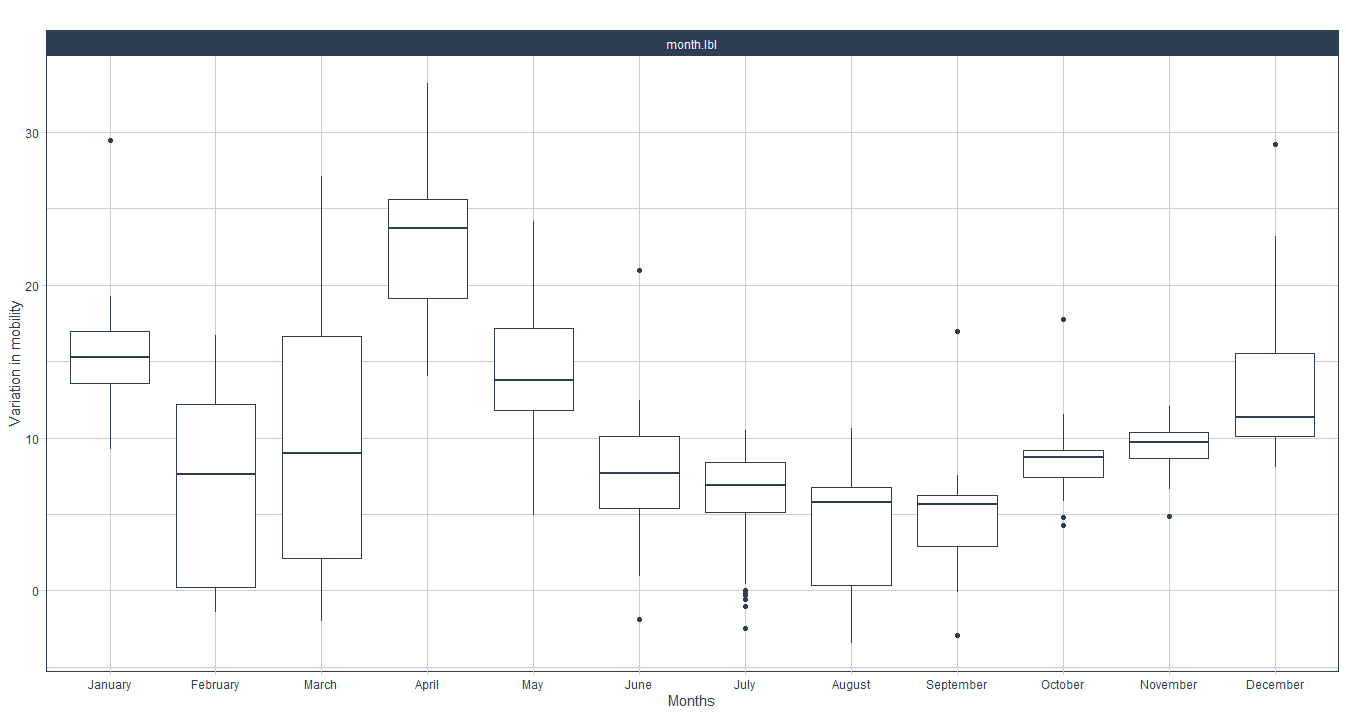

Supplement: Multimedia Appendix 1 [file publichealth_v10i1e46903_app1.zip › Figures 1-6 MA1/Figure_4/A/qc_g.png]

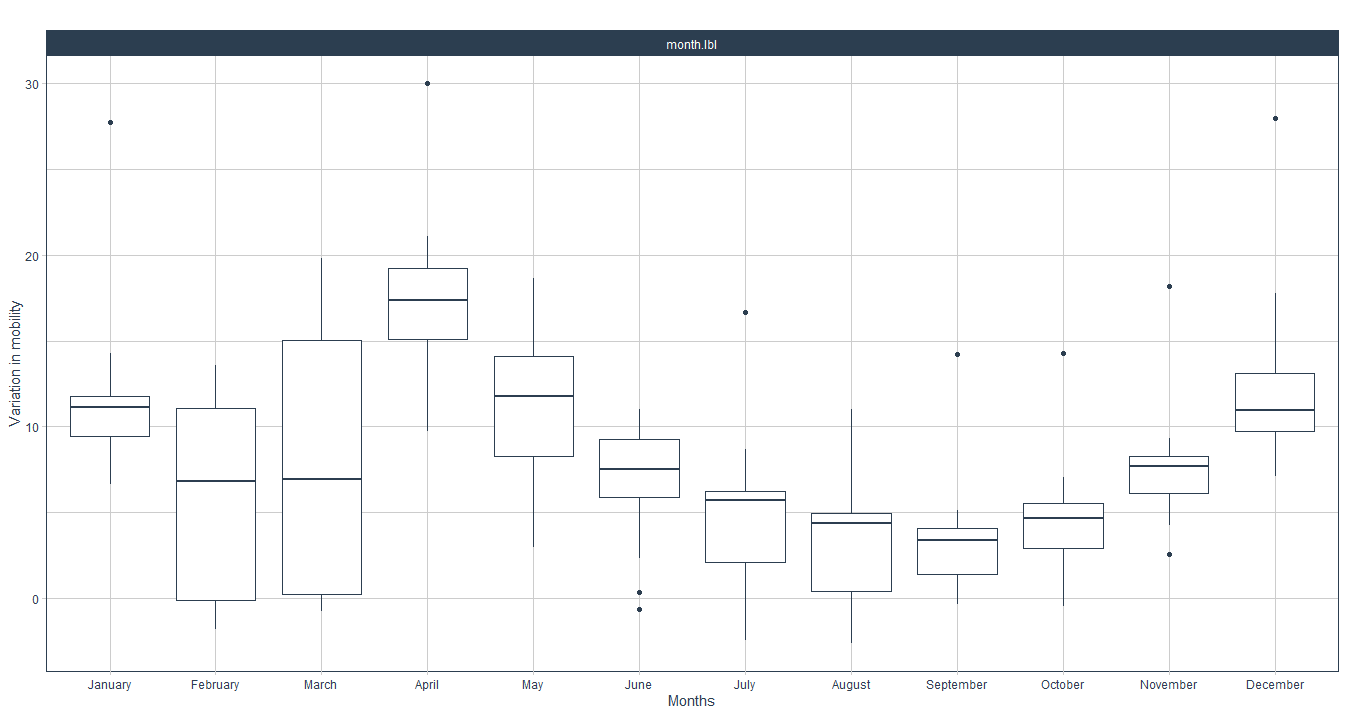

Supplement: Multimedia Appendix 1 [file publichealth_v10i1e46903_app1.zip › Figures 1-6 MA1/Figure_4/A/ab_g.png]

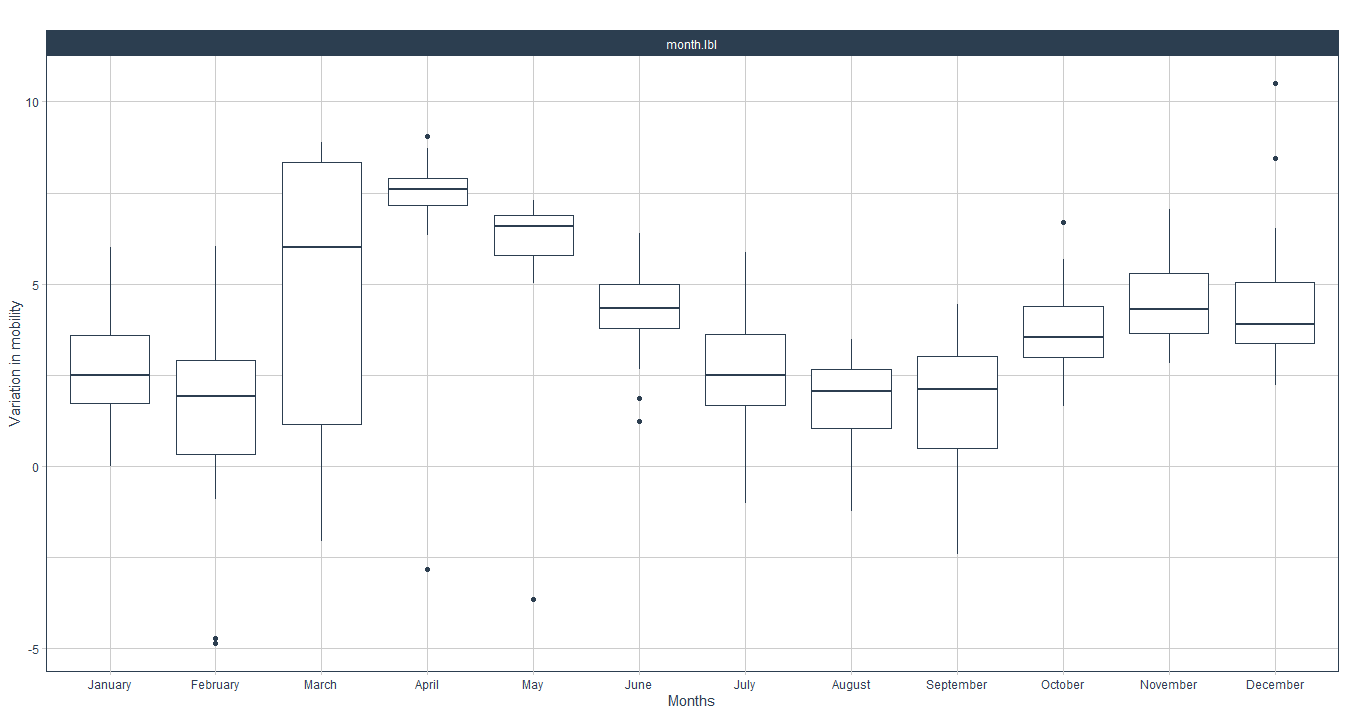

Supplement: Multimedia Appendix 1 [file publichealth_v10i1e46903_app1.zip › Figures 1-6 MA1/Figure_4/B/bc_e.png]

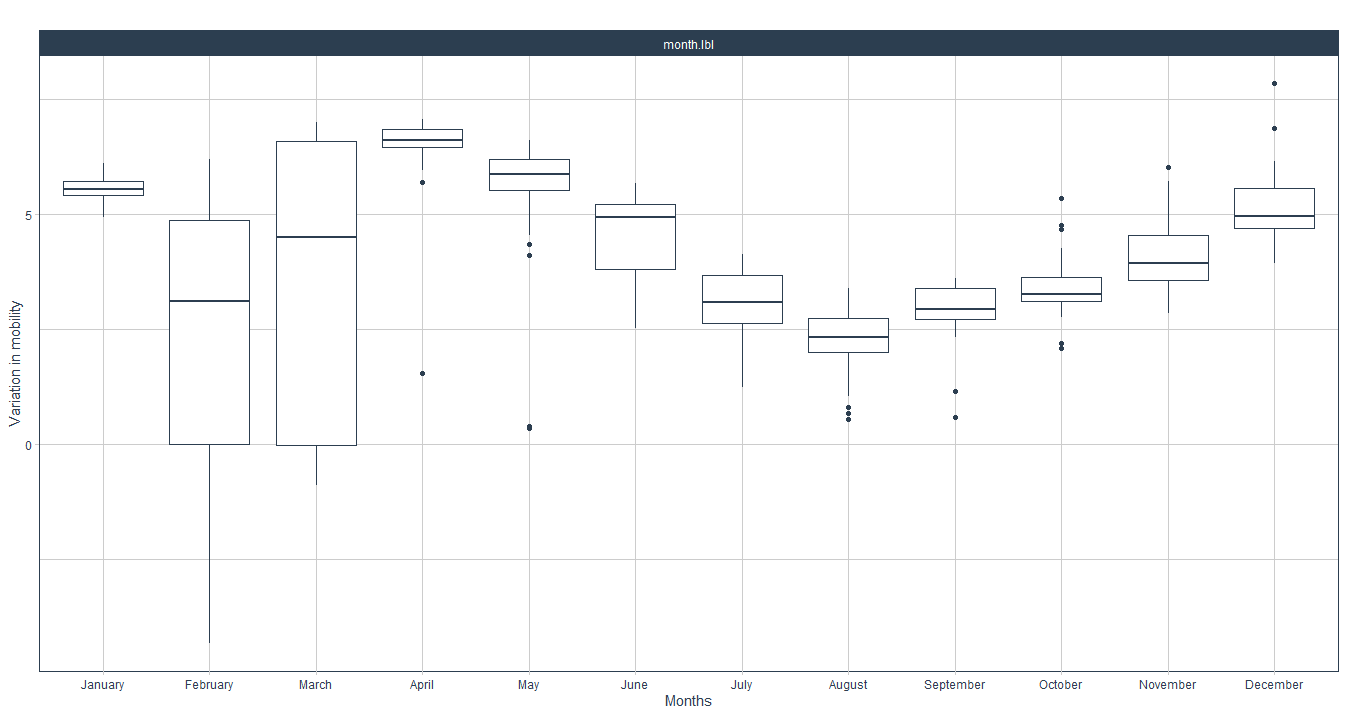

Supplement: Multimedia Appendix 1 [file publichealth_v10i1e46903_app1.zip › Figures 1-6 MA1/Figure_4/B/on_e.png]

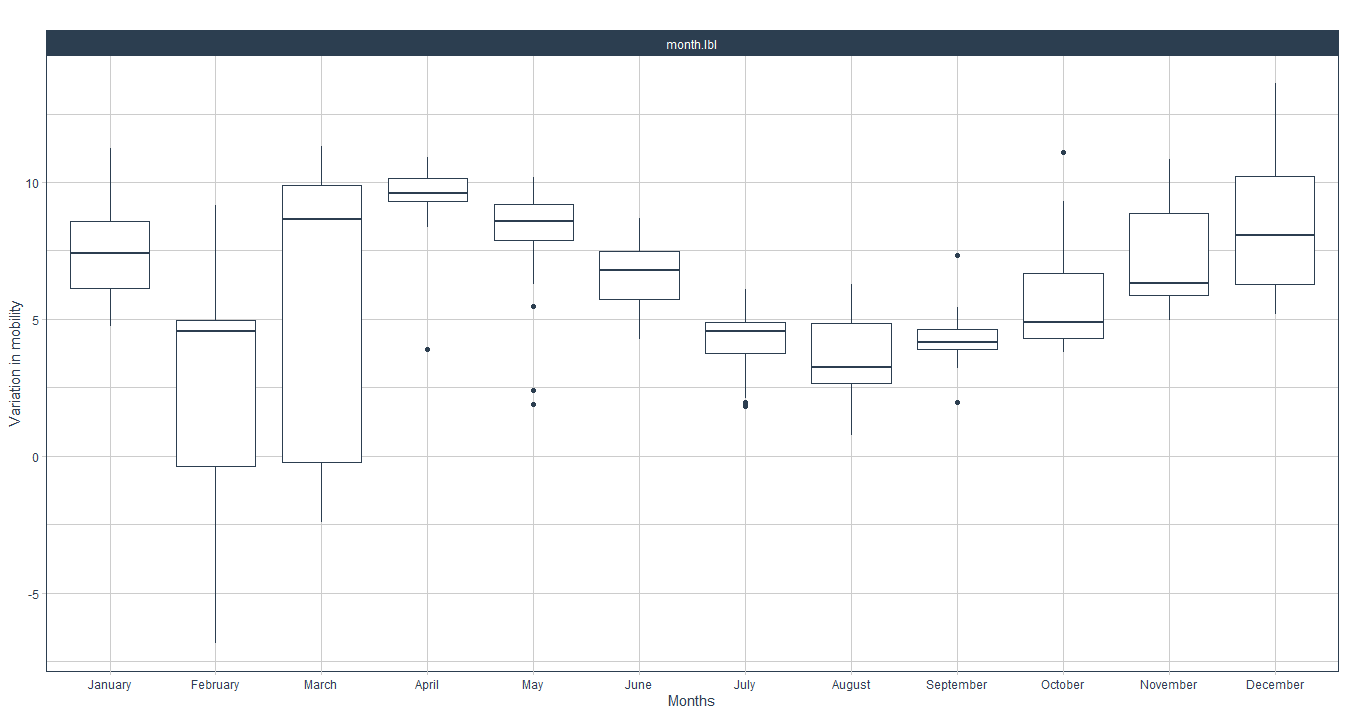

Supplement: Multimedia Appendix 1 [file publichealth_v10i1e46903_app1.zip › Figures 1-6 MA1/Figure_4/B/qc_e.png]

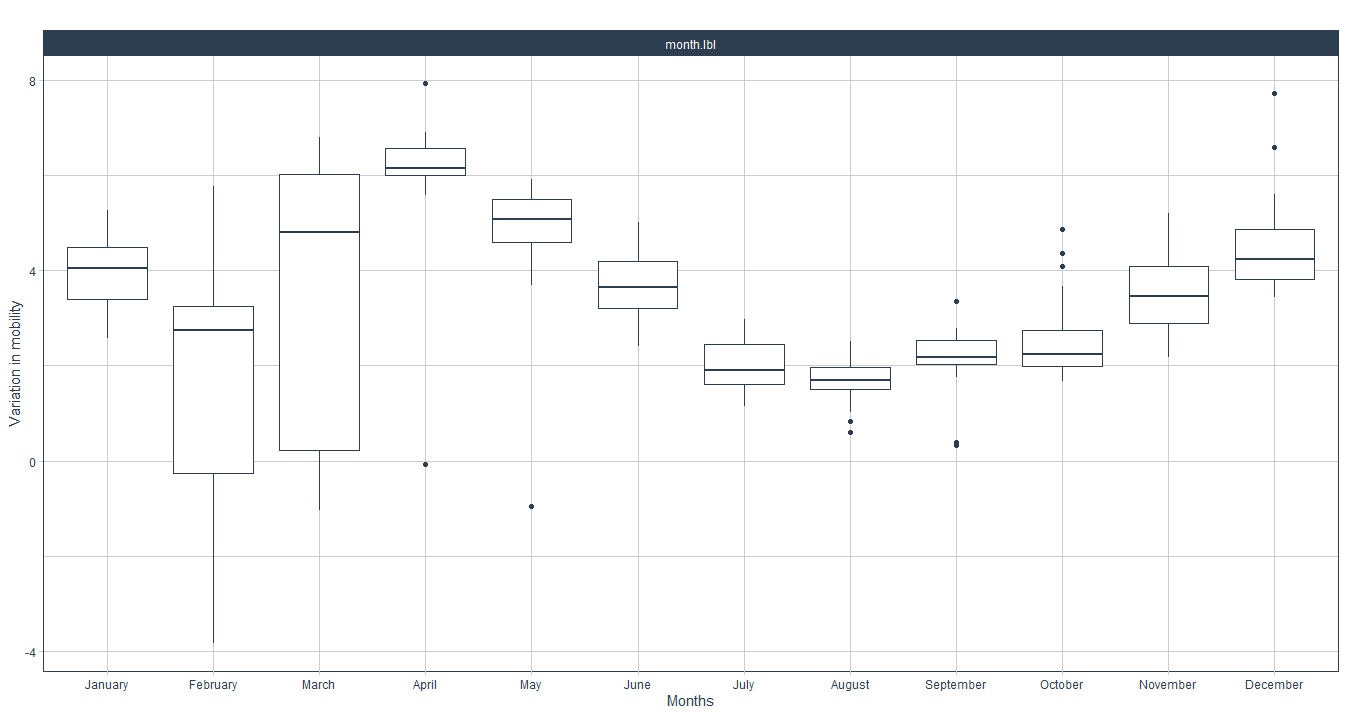

Supplement: Multimedia Appendix 1 [file publichealth_v10i1e46903_app1.zip › Figures 1-6 MA1/Figure_4/B/ab_e.png]
